# One-pot Ugi-azide and Heck reactions for the synthesis of heterocyclic systems containing tetrazole and 1,2,3,4-tetrahydroisoquinoline

**DOI:** 10.3762/bjoc.20.81

**Published:** 2024-04-23

**Authors:** Jiawei Niu, Yuhui Wang, Shenghu Yan, Yue Zhang, Xiaoming Ma, Qiang Zhang, Wei Zhang

**Affiliations:** 1 School of Pharmacy, Changzhou University, 1 Gehu Road, Changzhou 213164, China; 2 School of Chemistry and Life Sciences, Suzhou University of Science and Technology, 99 Xuefu Road, Suzhou 215009, China; 3 Department of Chemistry and Center for Green Chemistry, University of Massachusetts Boston, 100 Morrissey Blvd, Boston, MA 02125, USA

**Keywords:** Heck reaction, one-pot, tetrahydroisoquinoline, tetrazolo-pyrazino[2,1-*a*]isoquinolin-6(5*H*)-ones, tetrazole, Ugi-azide reaction

## Abstract

A new method for the synthesis of heterocyclic systems containing tetrazole and tetrahydroisoquinoline is developed via the performance of one-pot Ugi-azide and Heck cyclization reactions. The integration of the multicomponent and post-condensation reactions in one-pot maximizes the pot-, atom-, and step-economy (PASE).

## Introduction

**Figure 1:**
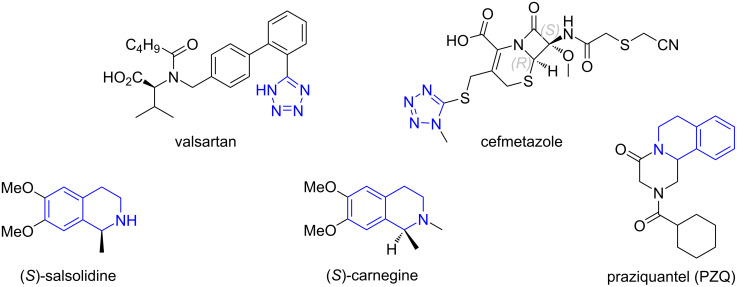
Representative bioactive tetrazole- and tetrahydroisoquinoline-containing compounds.

Tetrazole is a privileged heterocycle existing in a range of biological and medicinally interesting compounds [1–2] with antifungal [3–4], antibacterial [5], anticancer [6–7], antiparasitic [8], and antihypertensive properties [9] including FDA approved drugs such as valsartan and cefmetazole [10–11] (Figure 1). The tetrazole ring can also be found in functional materials for photography, imaging, and military applications [12–17]. The hydroisoquinoline core, such as 1,2,3,4-tetrahydroisoquinoline and pyrazino[2,1-*a*]isoquinolinone, is also a privileged heterocycle which can be found in natural products and synthetic compounds with antitumor, anti-HIV, antibiotic, antifungal, antivirus, and anti-inflammatory activities [18–21]. The antischistosomal drug praziquantel (PZQ), a tetrahydroisoquinoline derivative, is a commercialized drug for the treatment of schistosomiasis [22–25]. The combination of the privileged heterocycles tetrazole and tetrahydroisoquinoline in one molecule generates new molecules which could have biological activities.

**Scheme 1:**
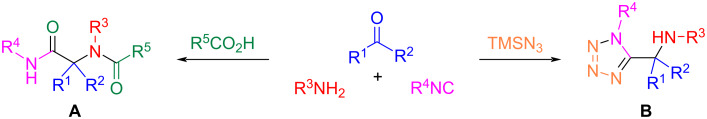
The Ugi and Ugi-azide reactions.

**Scheme 2:**
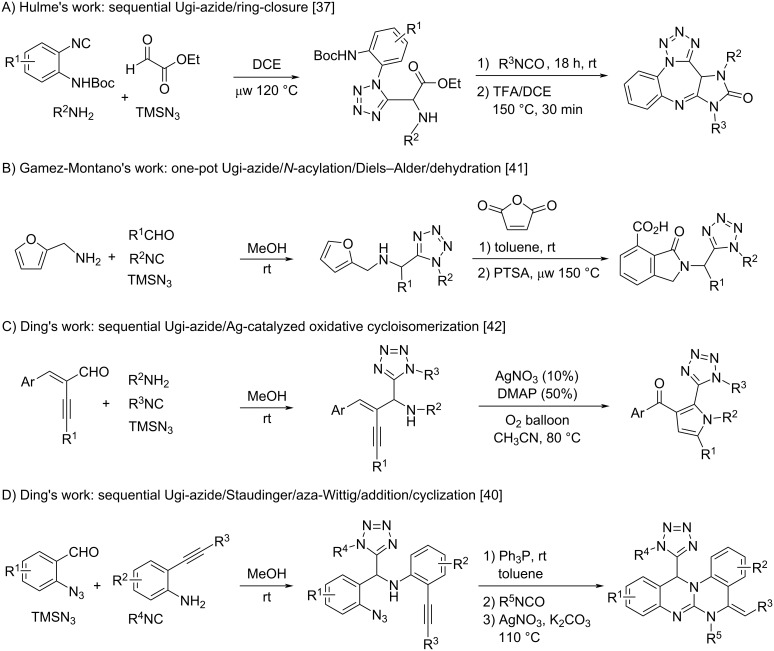
Ugi-azide and post-condensation reactions for the synthesis of various heterocyclic scaffolds.

A standard Ugi four-component reaction (Ugi-4CR) of an aldehyde, amine, isocyanide, and a carboxylic acid produces highly diverse peptidic structures **A** with up to four points of substitution (Scheme 1) [26–27]. By replacing the carboxylic acid with a nucleophilic azide reagent XN_3_ (generally TMSN_3_), the Ugi-azide four-component reaction (UA-4CR) of an aldehyde, amine, isocyanide, and azide gives 1,5-disubstituted 1*H*-tetrazoles (1,5-DS-1*H*-Ts) **B**. The performance of post-condensation reactions of UA-4CR adducts has resulted in various 1,5-DS-1*H*-Ts containing heterocyclic compounds [28–32], such as bis-heterocyclic lactam-tetrazoles [33–34], 2-tetrazolylmethyl-2,3,4,9-tetrahydro-1*H*-β-carbolines [35], ketopiperazinetetrazoles [36], imidazotetrazolodiazepinones [37], tetracyclic tetrazolylpyridoimidazoquinolines [38], bis-heterocyclic 1,5-disubstituted tetrazoleindolizines [39] and (*E*)-12-tetrazolyl-5*H*-quinazolino[3,2-*a*]quinazolines [40]. Among them, the Hulme group reported a UA-4CR/post-condensation sequence to give fused imidazotetrazolodiazepinones (Scheme 2A) [37]. The Gámez-Montaño group introduced a one-pot synthesis of Ugi-azide/*N*-acylation/Diels–Alder/dehydration reactions for isoindolin-1-one and 1,5-DS-T in a linked manner (Scheme 2B) [41]. The Ding group developed sequential Ugi-azide/Ag-catalyzed oxidative cycloisomerization reactions for the synthesis of 2-tetrazolyl-substituted 3-acylpyrroles (Scheme 2C) [42]. The Ding group also reported sequential Ugi-azide/Staudinger/aza-Wittig/addition/Ag-catalyzed cyclization reactions for obtaining 12-tetrazolyl-substituted (*E*)-5*H*-quinazolino[3,2-*a*]quinazolines (Scheme 2D) [40].

**Scheme 3:**
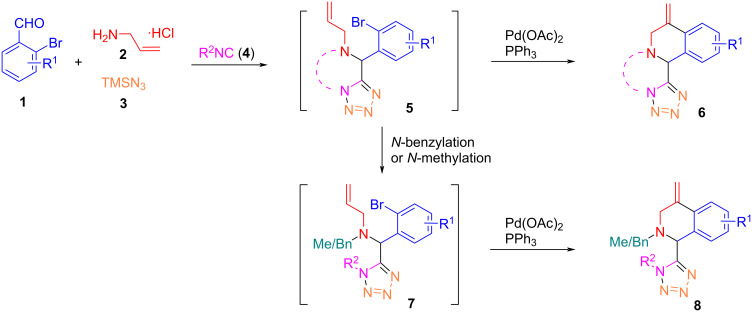
One-pot synthesis of tetrazolyl-1,2,3,4-tetrahydroisoquinoline.

There are numbers of Ugi and subsequential Heck (or reductive Heck) reactions that have been developed for the synthesis of poly-heterocyclic compounds [43–51]. Reported in this paper is a one-pot Ugi-azide reaction followed by an intramolecular Heck reaction for the synthesis of tetrazolyl-1,2,3,4-tetrahydroisoquinoline scaffolds **6** and **8** (Scheme 3). The first step is the Ugi-azide reaction of a 2-bromobenzoaldehyde **1**, allylamine hydrochloride (**2**), azidotrimethylsilane (TMSN_3_, **3**), and an isocyanide **4** affording tetrazoles **5**. If ethyl isocyanoacetate is used as the isocyanide source, the Ugi-azide reaction gives rise to ring-fused tetrazolo[1,5-*a*]pyrazin-6(5*H*)-one adducts **5**. The subsequent Pd-catalyzed intramolecular Heck reaction of compounds **5** or **7** then affords 1,2,3,4-tetrohydroisoquinolines **6** and **8**, respectively.

## Results and Discussion

Following the reported procedures [41], the Ugi-azide reaction of 2-bromobenzaldehyde (**1a**, 1 mmol), allylamine hydrochloride (**2**, 1 mmol), trimethylsilyl azide (**3**, 1 mmol) and *tert*-butyl isocyanide (**4a**, 1 mmol) in MeOH at 40 °C for 24 h afforded 1,5-DS-1*H*-T **5a** in 92% yield after chromatography purification. Our effort was then focused on the optimization of the intramolecular Heck reaction of **5a** for making 1,2,3,4-tetrahydroisoquinoline **6a**. A systematic evaluation of different catalysts and ligands, solvents, bases, as well as reaction temperatures and times was conducted (Table 1). The Heck reaction of **5a** was first examined by using 10 mol % Pd(OAc)_2_, 20 mol % PPh_3_, 2 equiv of Et_3_N in CH_3_CN or DMF at 105 °C for 24 h under N_2_ atmosphere. However, the reactions failed under these conditions (Table 1, entries 1 and 2). When K_2_CO_3_ was used as a base to replace Et_3_N, the reactions in either CH_3_CN or DMF for 3 h both gave cyclized product **6a** in 70% yield (Table 1, entries 3 and 4). An increase of the reaction time to 12 h did not improve the yield (Table 1, entry 5). The reaction was further evaluated in the absence of ligand which afforded the product in 35% yield (Table 1, entry 6). Screening of ligands, e.g., PCy_3_ and P(*o*-tol)_3_ reduced the yield of the desired product **6a** (Table 1, entries 7 and 8). Lowering the amount of Pd(OAc)_2_ or changing the reaction temperatures resulted low yields of **6a** (Table 1, entries 9–11). Similar results were observed from the reactions using other bases, such as K_3_PO_4_, NaOAc, and Cs_2_CO_3_ (Table 1, entries 12–14). Investigating other Pd catalysts, suche as PdCl_2_ and Pd(dba)_2_ also gave low yields (Table 1, entries 15 and 16). Since CH_3_CN is a more favorable solvent than DMF in green chemistry consideration [52–53], the optimal reaction conditions for the Heck reaction were to use 1 mmol of **5a** with 10 mol % Pd(OAc)_2_ and 20 mol % PPh_3_, 2 equiv of K_2_CO_3_ in 3 mL CH_3_CN at 105 °C for 3 h under N_2_ atmosphere which afforded product **6a** in 70% yield (Table 1, entry 3).

**Table 1:** Conditions for one-pot Ugi-azide and Heck reactions.^a^

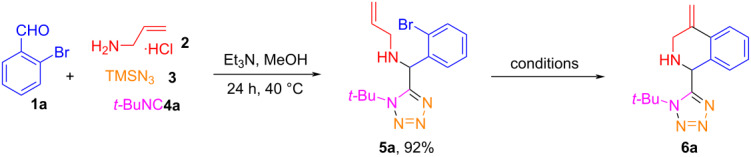							

Entry	Catalyst	Ligand	Solvent	Base	Temp (°C)	Time (h)	Yield (%)^b^

1	Pd(OAc)_2_	PPh_3_	MeCN	Et_3_N	105	24	–
2	Pd(OAc)_2_	PPh_3_	DMF	Et_3_N	105	24	–
3	Pd(OAc)_2_	PPh_3_	MeCN	K_2_CO_3_	105	3	70
4	Pd(OAc)_2_	PPh_3_	DMF	K_2_CO_3_	105	3	70
5	Pd(OAc)_2_	PPh_3_	MeCN	K_2_CO_3_	105	12	65
6	Pd(OAc)_2_	–	MeCN	K_2_CO_3_	105	6	35
7	Pd(OAc)_2_	PCy_3_	MeCN	K_2_CO_3_	105	6	46
8	Pd(OAc)_2_	P(*o*-tol)_3_	MeCN	K_2_CO_3_	105	6	56
9^c^	Pd(OAc)_2_	PPh_3_	MeCN	K_2_CO_3_	105	3	58
10	Pd(OAc)_2_	PPh_3_	MeCN	K_2_CO_3_	70	8	60
11	Pd(OAc)_2_	PPh_3_	MeCN	K_2_CO_3_	120	3	62
12	Pd(OAc)_2_	PPh_3_	MeCN	K_3_PO_4_	105	3	39
13	Pd(OAc)_2_	PPh_3_	MeCN	NaOAc	105	3	62
14	Pd(OAc)_2_	PPh_3_	MeCN	Cs_2_CO_3_	105	3	56
15	PdCl_2_	PPh_3_	MeCN	K_2_CO_3_	105	5	53
16	Pd(dba)_2_	PPh_3_	MeCN	K_2_CO_3_	105	6	61
**17** ^d^	**Pd(OAc)** **_2_**	**PPh** **_3_**	**MeCN**	**K** **_2_** **CO** **_3_**	**105**	**3**	**60**

^a^Reaction conditions: Ugi-azide step, 2-bromobenzaldehyde (**1a**, 1 mmol), allylamine hydrochloride (**2**, 1 mmol), trimethylsilyl azide (**3**, 1 mmol) and *tert*-butyl isocyanide (**4a**, 1 mmol), Et_3_N (1.2 mmol) in 5 mL MeOH, 40 °C for 24 h. Heck reaction step, catalyst (10 mol %), ligand (20 mol %), solvent (3 mL), base (2 equiv), nitrogen atmosphere. ^b^Isolated yield. ^c^Pd(OAc)_2_ 5 mol %, PPh_3_ 10 mol %. ^d^Reaction was carried out in one-pot, starting compound is **1a** (1 mmol), first Ugi-azide reaction followed by the Heck reaction.

The combination of an initial multicomponent reaction with post-condensation reactions in one-pot is a good strategy to develop high pot, atom and step economy (PASE) syntheses [54–58]. We then made the effort to integrate the Ugi and Heck reactions in one-pot for making tetrazolyl-1,2,3,4-tetrahydroisoquinolines **6**. Thus, a mixture of 2-bromobenzaldehyde (**1a**, 1 mmol), allylamine hydrochloride (**2**, 1 mmol), trimethylsilyl azide (**3**, 1 mmol), and *tert*-butyl isocyanide (**4a**, 1 mmol) was stirred in MeOH at 40 °C for 24 h, and after the reaction was completed, the solvent was evaporated under vacuum to give crude Ugi adduct **5a** which was used for the intramolecular Heck reaction without further purification. Thus, to the solution of crude **5a** dissolved in MeCN (3 mL) were added 10 mol % of Pd(OAc)_2_, 20 mol % of PPh_3_, and 2 equiv of K_2_CO_3_ and the mixture stirred for 3 h at 105 °C under N_2_ atmosphere to give the desired product **6a** in 60% isolated yield (entry 17 in Table 1).

**Scheme 4:**
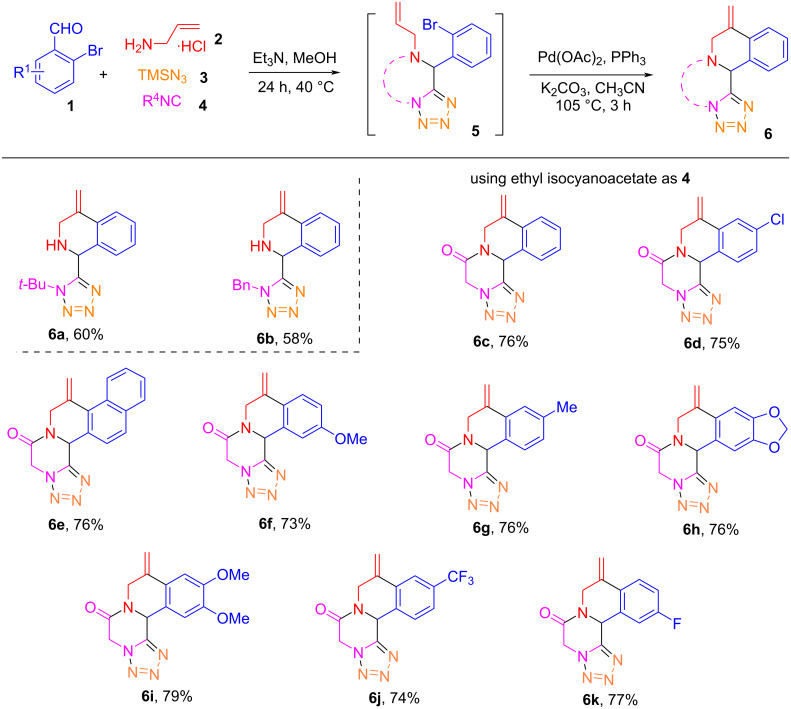
One-pot synthesis of tetrazolo-pyrazino[2,1-*a*]isoquinolin-6(5*H*)-ones **6**.

With the optimized one-pot reactions in hands, we next evaluated the substrate scope by synthesizing 11 derivatives (Scheme 4) using nine benzaldehydes **1**, two isonitriles or ethyl isocyanoacetate **4**, allylamine hydrochloride (**2**), and trimethylsilyl azide (**3**) for the initial Ugi-azide reaction. Among them, products **6a** and **6b** from the reaction of isonitriles were synthesized in moderate yields (58–60%). For the Ugi reaction involving isocyanoacetate, lactamination occurred spontaneously to provide the ring-fused tetrazolo[1,5-*a*]pyrazin-6(5*H*)-one adducts **5** which after intramolecular Heck reaction gave functionalized tetracyclic tetrazolo-pyrazino[2,1-*a*]isoquinolin-6(5*H*)-ones **6c**–**k** in 73–79% yields. The presence of electron-donating or electron-withdrawing groups on the aromatic ring did not show significant effects on the Heck reaction.

**Scheme 5:**
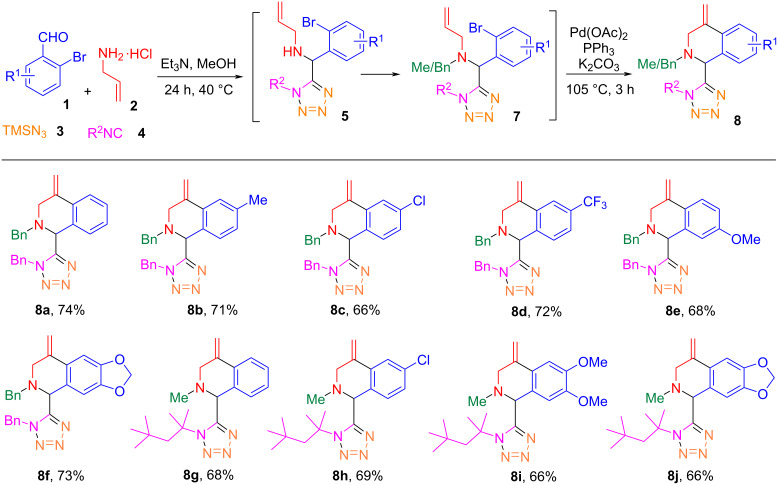
One-pot synthesis for tetrazolyl-1,2,3,4-tetrahydroisoquinolines **8**.

Products **6c**–**k** were obtained in higher yields than products **6a**,**b**. We believe that the secondary amine in the Ugi reaction products **5** could affect the yield of the Heck reaction. To address the issue, compounds **5** were *N*-alkylated to afford intermediates **7** which were used in the subsequent Heck reaction step. Thus, an alternative one-pot Ugi-azide/*N*-alkylation/Heck reaction procedure was developed (Scheme 5). A mixture of 2-bromobenzaldehyde (**1a**, 1 mmol), allylamine hydrochloride (**2**, 1 mmol), trimethylsilyl azide (**3**, 1 mmol) and benzyl isocyanide (1 mmol) in MeOH was reacted at 40 °C for 24 h. After evaporating the solvent, 3 mL CH_3_CN were added to the crude 1,5-DS-1*H*-T **5a** followed by the addition of 1 equiv of benzyl bromide and 2 equiv of K_2_CO_3_ for the alkylation reaction at 80 °C for 3 h to give *N*-benzylated compound **7a**. Finally, 10 mol % of Pd(OAc)_2_, 20 mol % of PPh_3_, 2 equiv of K_2_CO_3_ were added to the reaction mixture for the Heck reaction at 105 °C for 3 h under N_2_ atmosphere to afford tetrazolyl-1,2,3,4-tetrahydroisoquinoline **8a** in 74% isolated yield which is higher than the one-pot Ugi/Hecke reaction to give product **6b** (58%). Under the alternative one-pot reaction conditions involving an *N*-alkylation step, the substrate scope was explored by the preparation of 10 derivatives **8a**–**j** (Scheme 5) using seven benzaldehydes **1**, two isonitriles **4**, and allylamine hydrochloride (**2**) with trimethylsilyl azide (**3**) for the Ugi-azide reaction. The *N*-alkylations were conducted using benzyl bromide and iodomethane, respectively. The final products **8b**–**j** were obtained in 66–74% yields.

**Scheme 6:**
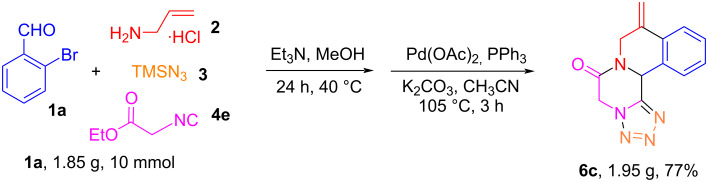
Gram-scale two-step one-pot synthesis of **6c**.

To evaluate the scalability of the two-step one-pot reaction protocol, we performed the synthesis of tetracyclic tetrazolo-pyrazino[2,1-*a*]isoquinolin-6(5*H*)-one **6c** in gram quantity from 10 mmol of **1a** which led to the formation of product **6c** in a satisfactory yield 77% (Scheme 6).

**Figure 2:**
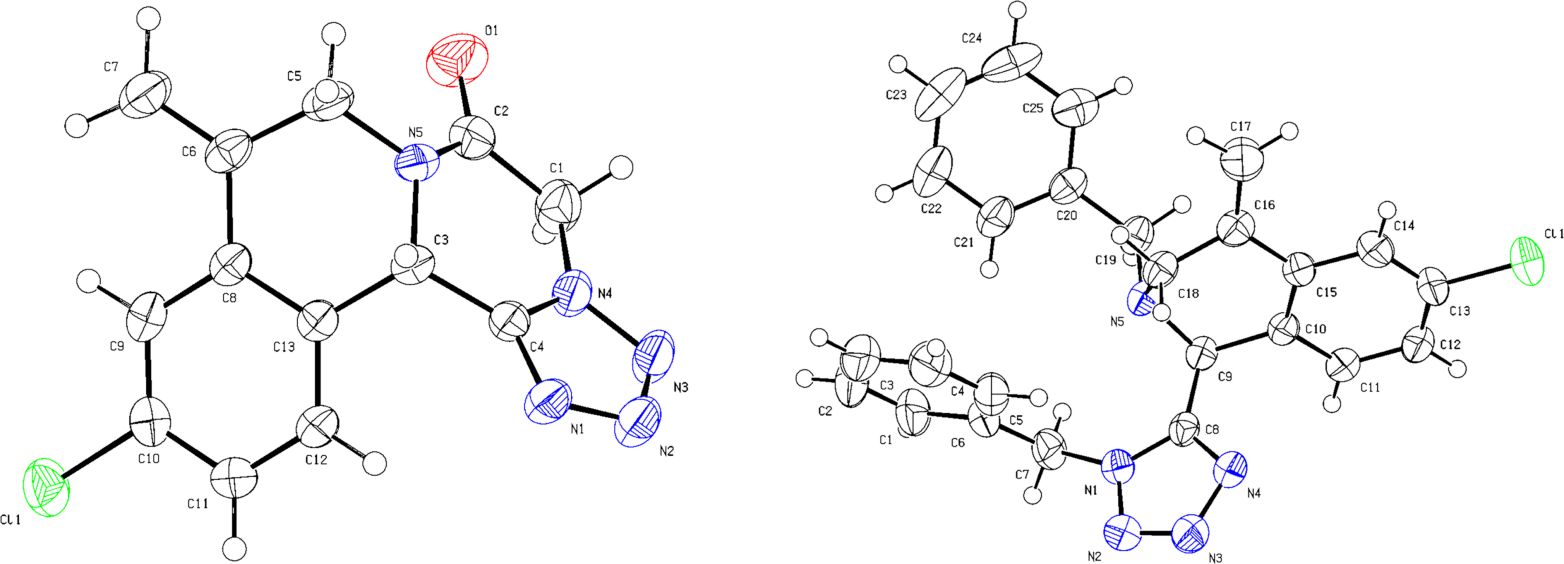
ORTEP diagrams of compound **6d** (left) [CCDC: 2164364] and **8c** (right) [CCDC: 2321622].

The products **6** and **8** were characterized by ^1^H and ^13^C NMR, and HRMS analysis. In addition, single crystals of compound **6d** and **8c** were obtained for X-ray analysis to confirm the structures (Figure 2).

## Conclusion

In conclusion, we have developed a one-pot synthesis with two or three steps for making tetrazolo-pyrazino[2,1-*a*]isoquinolin-6(5*H*)-ones. The initial Ugi-azide four-component reaction constructs the tetrazole motif while the subsequent intramolecular Heck reaction assembles the tetrahydroisoquinoline. The one-pot reaction avoids the intermediate purification which has favorable PASE in the synthesis of heterocyclic compounds.

## Experimental

### General procedure for the synthesis of Ugi-azide adduct **5a**

A solution of 2-bromobenzaldehyde **1** (1 mmol, 1 equiv), allylamine hydrochloride (**2**, 1 mmol, 1 equiv), trimethylsilyl azide (**3**, 1 mmol, 1 equiv) and *tert*-butyl isocyanide **4a** (1 mmol, 1 equiv) in MeOH (5 mL) with Et_3_N (1.5 mmol) was heated at 40 °C for 24 h in a sealed vial. Upon completion of the reaction, the reaction mixture was filtered and evaporated under vacuum to give crude products **5a**. Further purification was conducted by flash chromatography with 1:6 petroleum ether/EtOAc to afford **5a** in 92% yields. The adduct was confirmed by NMR.

### General procedure for the Heck reaction; synthesis of product **6a**

A mixture of Ugi-azide adduct **5a** (1 mmol), Pd(OAc)_2_ (0.1 mmol), PPh_3_ (0.2 mmol), K_2_CO_3_ (2 mmol) or NaOAc (2 mmol) in MeCN (3 mL) was stirred at 105 °C for 3 h under nitrogen atmosphere. After aqueous work-up, the crude product was purified by flash chromatography with 1:4 ethyl acetate*/*petroleum ether to afford product **6a**.

### General procedure for the one-pot synthesis of tetrazole-containing 1,2,3,4-tetrahydroisoquinolines **6**

A mixture of 2-bromobenzaldehyde **1** (1 mmol), allylamine hydrochloride (**2**, 1 mmol), trimethylsilyl azide (**3**, 1 mmol) and isocyanide **4** (1 mmol) in MeOH was stirred at 40 °C for 24 h. After the reaction was complete, the solvent was evaporated under vacuum to give the crude Ugi adduct **5**, which was used in the Heck reaction without further purification. To a solution of the crude intermediate **5** in MeCN (3 mL) was added 10 mol % of Pd(OAc)_2_, 20 mol % of PPh_3_, 2 equiv of K_2_CO_3_ and the mixture stirred for 3 h at 105 °C under N_2_ atmosphere. After aqueous work-up, the crude product was purified by flash chromatography with 1:3 ethyl acetate*/*petroleum ether to afford products **6**.

### General procedure for the one-pot synthesis of tetrazolyl-1,2,3,4-tetrahydroisoquinolines **8**

A mixture of 2-bromobenzaldehyde **1** (1 mmol), allylamine hydrochloride (**2**, 1 mmol), trimethylsilyl azide (**3**, 1 mmol) and isocyanide **4** (1 mmol) in MeOH was reacted at 40 °C for 24 h. After evaporating the solvent, 3 mL CH_3_CN were added to the crude 1,5-DS-1*H*-T **5** followed by the addition of 1 equiv of benzyl bromide or iodomethane and 2 equiv of K_2_CO_3_ for the alkylation reaction at 80 °C for 3 h to give *N*-alkylated compounds **7**. Finally, 10 mol % of Pd(OAc)_2_, 20 mol % of PPh_3_, 2 equiv of K_2_CO_3_ were added to the reaction mixture for the Heck reaction at 105 °C for 3 h under N_2_ atmosphere. After aqueous work-up, the crude products were purified by flash chromatography with 1:4 ethyl acetate*/*petroleum ether to afford products **8**.

## Supporting Information

File 1 General reaction procedures, compound characterization data, and copies of NMR spectra.

File 2 Crystallographic information file for compound **6d**.

File 3 Crystallographic information file for compound **8c**.

## Data Availability

All data that supports the findings of this study is available in the published article and/or the supporting information to this article. Data generated and analyzed during this study is openly available in CCDC. The data of CCDC-2164364 can be obtained free of charge at doi: https://doi.org/10.5517/ccdc.csd.cc2bn66f; the data of CCDC- 2321622: can be obtained free of charge at doi: https://doi.org/10.5517/ccdc.csd.cc2hxv1b.
