# Supplementary material for: One-pot Ugi-azide and Heck reactions for the synthesis of heterocyclic systems containing tetrazole and 1,2,3,4-tetrahydroisoquinoline
Source: Beilstein J Org Chem. 2024 Apr 23;20:912–20. doi: 10.3762/bjoc.20.81 (PMC11070971; doi:10.3762/bjoc.20.81)
Supplement: File 1 — General reaction procedures, compound characterization data, and copies of NMR spectra. [file Beilstein_J_Org_Chem-20-912-s001.pdf]

BEILSTEIN JOURNAL OF ORGANIC CHEMISTRY

# Supporting Information

for

# One-pot Ugi-azide and Heck reactions for the synthesis of heterocyclic systems containing tetrazole and 1,2,3,4-tetrahydroisoquinoline

Jiawei Niu, Yuhui Wang, Shenghu Yan, Yue Zhang, Xiaoming Ma, Qiang Zhang and Wei Zhang

*Beilstein J. Org. Chem.* **2024,** *20,* 912–920. doi:10.3762/bjoc.20.81

# General reaction procedures, compound characterization data, and copies of NMR spectra

License and Terms: This is a supporting information file under the terms of the Creative Commons Attribution License (https://creativecommons.org/licenses/by/4.0). Please note that the reuse, redistribution and reproduction in particular requires that the author(s) and source are credited and that individual graphics may be subject to special legal provisions.
The license is subject to the *Beilstein Journal of Organic Chemistry* terms and conditions: (https://www.beilstein-journals.org/bjoc/terms)

**Table of contents**

1. General information ........................................ S1

2. Analytical data of products ........................................ S1

3. X-ray report of **6d** and **8c** ........................................ S6

4. $^{1}H$ NMR, $^{13}C$ NMR of products **5a**, **6** and **8** ........................................ S8

## 1. General information

Chemicals and solvents were purchased from commercial source and used without further purification. $^{1}$H (300 MHz, 400 MHz), $^{13}$C NMR spectra (75 MHz or 126 MHz) were recorded on a Bruker NMR spectrometer. LC–MS were performed on an Agilent 2100 system with C18 column (5.0 µm, 6.0 × 50 mm). The mobile phases were MeOH and $H_2O$ both containing 0.05% trifluoroacetic acid. A linear gradient was used to increase from 25:75 (v/v) MeOH/$H_2O$ to 100% MeOH in 7.0 min at a flow rate of 0.7 mL/min. UV detections were conducted at 210 nm, 254 nm, and 365 nm. Low resolution mass spectra were recorded in APCI (atmospheric pressure chemical ionization). HRMS were performed on Agilent 6540 Q-TOF mass spectrometer (ESI). Flash column chromatography was performed using silica gel (200–300 mesh).

## 2. Analytical data of products

*N-((2-Bromophenyl)(1-(tert-butyl)-1H-tetrazol-5-yl)methyl)prop-2-en-1-amine(5a)*

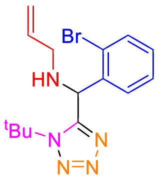

**5a**

White solid, 321mg, 92% yield. $^{1}$H NMR (400 MHz, Chloroform-*d*) δ 7.59 (dd, *J* = 7.9, 1.5 Hz, 1H), 7.20 (dd, *J* = 7.5, 1.4 Hz, 1H), 7.16 – 7.11 (m, 1H), 7.08 (dd, *J* = 7.6, 1.8 Hz, 1H), 5.94 (dddd, *J* = 18.4, 9.5, 7.2, 5.4 Hz, 1H), 5.72 (s, 1H), 5.10 (t, *J* = 1.5 Hz, 1H), 5.07 (dd, *J* = 6.2, 1.6 Hz, 1H), 3.42 – 3.32 (m, 1H), 3.19 – 3.12 (m, 1H), 2.36 (s, 1H), 1.57 (s, 9H). $^{13}$C NMR (101 MHz, Chloroform-*d*) δ 154.7, 138.2, 136.0, 133.6, 130.0, 129.0, 128.3, 124.3, 117.6, 61.6, 56.2, 50.9, 29.8. HRMS (ESI) calcd for $C_{15}H_{20}BrN_5$ ([M+Na]$^+$): 372.0800, found 372.0792.

*1-(1-(tert-Butyl)-1H-tetrazol-5-yl)-4-methylene-1,2,3,4-tetrahydroisoquinoline(6a)*

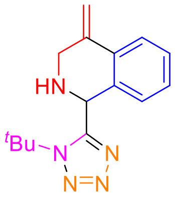

**6a**

Light yellow oil, 161mg, 60% yield. $^{1}$H NMR (400 MHz, Chloroform-*d*) δ 7.73 (dd, *J* = 8.0, 1.4 Hz, 1H), 7.29 (d, *J* = 7.5 Hz, 1H), 7.17 (d, *J* = 1.3 Hz, 1H), 6.66 (d, *J* = 7.7 Hz, 1H), 5.74 (s, 1H), 5.61 (s, 1H), 5.04 (s, 1H), 3.80 (d, *J* = 15.2 Hz, 1H), 3.68 (s, 1H), 2.03 (d, *J* = 16.7 Hz, 1H), 1.85 (s, 9H). $^{13}$C NMR (101 MHz, Chloroform-*d*) δ 155.5, 138.8, 133.9, 133.0, 128.2, 128.0, 126.7, 124.5, 108.3, 62.1, 53.1, 48.6, 30.2. HRMS (ESI) calcd for $C_{15}H_{19}N_5$ ([M+Na]$^+$): 292.1538, found 292.1520.

*1-(1-Benzyl-1H-tetrazol-5-yl)-4-methylene-1,2,3,4-tetrahydroisoquinoline(6b)*

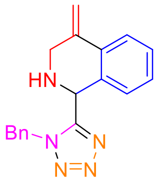

**6b**

yellow oil, 174 mg, 58 yield. $^{1}$H NMR (300 MHz, Chloroform-*d*) δ 7.67 (dd, *J* = 8.0, 1.3 Hz, 1H), 7.21 (dd, *J* = 5.2, 2.2 Hz, 4H), 7.09 – 7.03 (m, 2H), 6.99 (td, *J* = 7.6, 1.3 Hz, 1H), 6.52 (d, *J* = 7.8 Hz, 1H), 5.66 – 5.55 (m, 3H), 5.48 (d, *J* = 14.9 Hz, 1H), 5.04 (d, *J* = 1.4 Hz, 1H), 3.68 – 3.54 (m, 2H), 2.47 (s, 1H). $^{13}$C NMR (101 MHz, DMSO-$d_6$) δ 150.4, 133.5, 128.5, 127.6, 126.9, 123.5, 123.2, 123.0, 122.9, 122.7, 121.9, 119., 103.2, 72.4, 72.1, 71.8, 47.1, 46.2, 43.6. HRMS (ESI) calcd for $C_{18}H_{17}N_5$ ([M+Na]$^+$): 326.1832, found 326.1817.

*9-Methylene-9,13b-dihydro-8H-tetrazolo[5',1':3,4]pyrazino[2,1-a]isoquinolin-6(5H)-one (6c)*

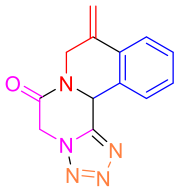

**6c**

Light yellow solid, 192 mg, 76% yield. $^{1}$H NMR (400 MHz, Chloroform-d) δ 7.75 (d, J = 7.6 Hz, 1H), 7.70 (d, J = 7.7 Hz, 1H), 7.34 (dt, J = 15.8, 7.3 Hz, 2H), 6.33 (s, 1H), 5.75 (s, 1H), 5.43 (d, J = 15.0 Hz, 1H), 5.34 (s, 1H), 5.20 (d, J = 18.0 Hz, 1H), 4.98 (d, J = 18.0 Hz, 1H), 3.91 (d, J = 15.0 Hz, 1H). $^{13}$C NMR (126 MHz, Chloroform-*d*) δ 159.5, 147.9, 135.5, 132.57, 129.8, 129.2, 129.0, 125.9, 124.9, 112.0, 53.7, 48.0, 47.8. HRMS (ESI) calcd for $C_{13}H_{11}N_5O$ ([M+Na]$^+$): 276.0866, found 276.0861.

*11-Chloro-9-methylene-9,13b-dihydro-8H-tetrazolo[5',1':3,4]pyrazino[2,1-a]isoquinolin-6(5H)-one (6d)*

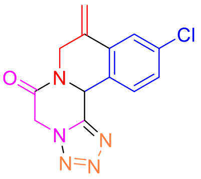


**6d**

White solid, 215 mg, 75% yield. $^{1}$H NMR (400 MHz, Chloroform-d) δ 7.78 (d, J = 8.5 Hz, 1H), 7.68 (d, J = 1.9 Hz, 1H), 7.31 (dd, J = 8.5, 1.9 Hz, 1H), 6.31 (s, 1H), 5.77 (s, 1H), 5.45 (d, J = 15.1 Hz, 1H), 5.42 (s, 1H), 5.24 (d, J = 18.0 Hz, 1H), 5.03 (d, J = 18.0 Hz, 1H), 3.90 (d, J = 15.0 Hz, 1H). $^{13}$C NMR (126 MHz, Chloroform-*d*) δ 159.4, 147.7, 135.4, 134.5, 134.3, 129.0, 128.1, 127.6, 124.9, 113.3, 53.3, 47.8, 47.6. HRMS (ESI) calcd for $C_{13}H_{10}ClN_5O$ ([M+Na]$^+$): 310.0472, found 310.0472.

*9-Methylene-9,15b-dihydro-8H-benzo[g]tetrazolo[5',1':3,4]pyrazino[2,1-a]isoquinolin-6(5H)-one (6e)*

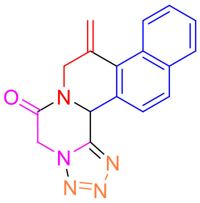

**6e**

Light yellow solid, 230 mg, 76% yield. $^{1}$H NMR (400 MHz, Chloroform-*d*) δ 8.38 (d, *J* = 8.0 Hz, 1H), 7.91 – 7.85 (m, 1H), 7.78 (d, *J* = 8.6 Hz, 1H), 7.62 – 7.52 (m, 2H), 7.34 (d, *J* = 8.6 Hz, 1H), 6.15 (s, 1H), 5.89 (s, 1H), 5.75 (d, *J* = 1.9 Hz, 1H), 5.24 (d, *J* = 17.9 Hz, 1H), 5.01 (dd, *J* = 16.5, 7.9 Hz, 2H), 4.30 (d, *J* = 15.0 Hz, 1H). $^{13}$C NMR (126 MHz, Chloroform-*d*) δ 159.2, 147.3, 134.9, 134.1, 132.9, 130.0, 129.5, 128.8, 128.7, 127.5, 126.9, 124.9, 120.6, 120.2, 52.6, 50.0, 47.8. HRMS (ESI) calcd for $C_{17}H_{13}N_5O$ ([M+Na]$^+$): 326.1011, found 326.1018.

*12-Methoxy-9-methylene-9,13b-dihydro-8H-tetrazolo[5',1':3,4]pyrazino[2,1-a]isoquinolin-6(5H)-one (6f)*

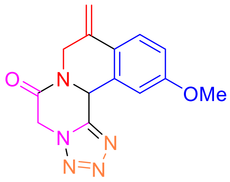


**6f**

yellow solid, 206 mg, 73% yield. $^{1}$H NMR (500 MHz, Chloroform-*d*) δ 7.60 (d, *J* = 8.8 Hz, 1H), 7.30 (d, *J* = 2.0 Hz, 1H), 6.85 (dd, *J* = 8.8, 2.6 Hz, 1H), 6.28 (s, 1H), 5.58 (d, *J* = 1.5 Hz, 1H), 5.38 (d, *J* = 14.9 Hz, 1H), 5.19 (s, 1H), 5.18 – 5.13 (m, 1H), 4.97 (dd, *J* = 17.9, 1.4 Hz, 1H), 3.85 (d, *J* = 14.9 Hz, 1H), 3.77 (s, 3H). $^{13}$C NMR (126 MHz, Chloroform-*d*) δ 160.0, 159.4, 147.9, 135.0, 131.0, 126.3, 124.9, 115.7, 110.3, 109.5, 55.5, 53.8, 48.1, 47.7. HRMS (ESI) calcd for $C_{14}H_{13}N_5O_2$ ([M+Na]$^+$): 306.0979, found 306.0967.

*11-Methyl-9-methylene-9,13b-dihydro-8H-tetrazolo[5',1':3,4]pyrazino[2,1-a]isoquinolin-6(5H)-one (6g)*

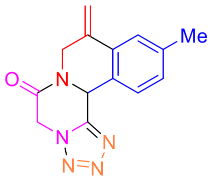


**6g**

brown solid, 202 mg, 76% yield. $^{1}$H NMR (400 MHz, Chloroform-*d*) δ 7.63 (d, *J* = 8.1 Hz, 1H), 7.51 (s, 1H), 7.13 (d, *J* = 8.1 Hz, 1H), 6.29 (s, 1H), 5.74 (s, 1H), 5.42 (d, *J* = 15.0 Hz, 1H), 5.32 (s, 1H), 5.20 (d, *J* = 18.0 Hz, 1H), 4.97 (d, *J* = 17.9 Hz, 1H), 3.89 (d, *J* = 15.0 Hz, 1H), 2.35 (s, 3H). $^{13}$C NMR (126 MHz, Chloroform-*d*) δ 159.5, 148.1, 139.1, 135.6, 132.3, 129.8, 127.0, 125.8, 125.3, 111.7, 53.6, 48.1, 47.8, 21.2. HRMS (ESI) calcd for $C_{14}H_{13}N_5O$ ([M+Na]$^+$): 290.1023, found 290.1018.

*9-Methylene-9,14b-dihydro-8H-[1,3]dioxolo[4,5-g]tetrazolo[5',1':3,4]pyrazino[2,1-a]isoquinolin-6(5H)-one (6h)*

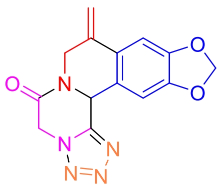

**6h**

brown solid, 225 mg, 76% yield. $^{1}$H NMR (500 MHz, Chloroform-*d*) δ 7.28 (s, 1H), 7.10 (s, 1H), 6.24 (s, 1H), 5.99 (d, *J* = 1.1 Hz, 1H), 5.94 (d, *J* = 1.1 Hz, 1H), 5.55 (d, *J* = 1.3 Hz, 1H), 5.39 (d, *J* = 14.9 Hz, 1H), 5.24 (s, 1H), 5.20 (d, *J* = 18.1 Hz, 1H), 4.99 (dd, *J* = 18.0, 1.3 Hz, 1H), 3.84 (d, *J* = 14.9 Hz, 1H). $^{13}$C NMR (126 MHz, Chloroform-*d*) δ 159.5, 148.5, 148.5, 148.0, 135.3, 127.0, 123.9, 110.4, 106.1, 104.5, 101.8, 53.6, 47.9, 47.7. HRMS (ESI) calcd for $C_{14}H_{11}N_5O_3$ ([M+Na]$^+$): 320.0753, found 320.0760.

*11,12-Dimethoxy-9-methylene-9,13b-dihydro-8H-tetrazolo[5',1':3,4]pyrazino[2,1-a]isoquinolin-6(5H)-one (6i)*

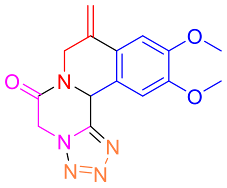

**6i**

brown solid, 247 mg, 79% yield. $^{1}$H NMR (500 MHz, Chloroform-*d*) δ 7.40 (s, 1H), 7.11 (s, 1H), 6.31 (s, 1H), 5.59 (d, *J* = 1.5 Hz, 1H), 5.43 (d, *J* = 14.8 Hz, 1H), 5.25 (s, 1H), 5.23 – 5.18 (m, 1H), 5.02 (dd, *J* = 18.0, 1.4 Hz, 1H), 3.90 (s, 3H), 3.86 (s, 3H), 3.84 (d, *J* = 14.8 Hz, 1H). $^{13}$C NMR (126 MHz, Chloroform-*d*) δ 159.4, 149.9, 149.4, 148.2, 135.2, 125.0, 122.4, 109.5, 108.4, 106.8, 56.1, 56.0, 53.6, 48.1, 47.7. HRMS (ESI) calcd for $C_{15}H_{15}N_5O_3$ ([M+Na]$^+$): 336.1069, found 336.1069.

*9-Methylene-11-(trifluoromethyl)-9,13b-dihydro-8H-tetrazolo[5',1':3,4]pyrazino[2,1-a]isoquinolin-6(5H)-one (6j)*

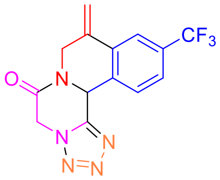


**6j**

White solid, 237 mg, 74% yield. $^{1}$H NMR (500 MHz, Chloroform-*d*) δ 8.02 (d, *J* = 8.3 Hz, 1H), 7.95 (s, 1H), 7.59 (d, *J* = 8.3 Hz, 1H), 6.38 (s, 1H), 5.86 (s, 1H), 5.53 – 5.48 (m, 2H), 5.26 (d, *J* = 18.4 Hz, 1H), 5.04 (dd, *J* = 18.1, 1.2 Hz, 1H), 3.93 (d, *J* = 15.0 Hz, 1H). $^{13}$C NMR (126 MHz, Chloroform-*d*) δ 159.4, 147.5, 134.4, 133.5, 133.1, 131.8, 131.6, 127.0, 125.5, 121.9, 113.9, 53.5, 47.8, 47.7. $^{19}$F NMR (282 MHz, Chloroform-*d*) δ -63.0. HRMS (ESI) calcd for $C_{14}H_{10}F_3N_5O$ ([M+Na]$^+$): 344.0759, found 344.0735.

*12-Fluoro-9-methylene-9,13b-dihydro-8H-tetrazolo[5',1':3,4]pyrazino[2,1-a]isoquinolin-6(5H)-one (6k)*

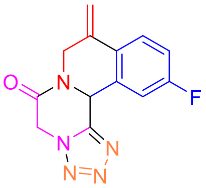


**6k**

Light yellow solid, 208 mg, 77% yield. $^{1}$H NMR (500 MHz, Chloroform-*d*) δ 7.70 (dd, *J* = 8.8, 5.5 Hz, 1H), 7.59 (dd, *J* = 9.4, 2.4 Hz, 1H), 7.08 (td, *J* = 8.4, 2.6 Hz, 1H), 6.32 (s, 1H), 5.69 (s, 1H), 5.45 (d, *J* = 15.0 Hz, 1H), 5.34 (s, 1H), 5.24 (d, *J* = 18.0 Hz, 1H), 5.06 – 4.99 (m, 1H), 3.90 (d, *J* = 15.0 Hz, 1H). $^{13}$C NMR (126 MHz, Chloroform-*d*) δ 163.6, 161.6, 159.4, 147.6, 134.5, 131.7, 128.7, 127.0, 127.0, 116.7, 116.6, 113.4, 113.2, 111.8, 53.5, 47.9, 47.8. $^{19}$F NMR (282 MHz, Chloroform-*d*) δ -109.9. HRMS (ESI) calcd for $C_{13}H_{10}FN_5O$ ([M+Na]$^+$): 294.0771, found 294.0767.

*2-Benzyl-1-(1-benzyl-1H-tetrazol-5-yl)-4-methylene-1,2,3,4-tetrahydroisoquinoline (**8a**)*

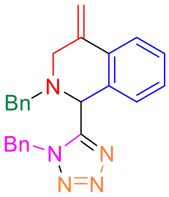


**8a**

Light yellow solid, 290 mg, 74% yield. $^{1}$H NMR (400 MHz, Chloroform-*d*) δ 7.74 – 7.66 (m, 1H), 7.37 – 7.29 (m, 3H), 7.25 – 7.14 (m, 6H), 7.03 (d, *J* = 1.3 Hz, 1H), 6.86 – 6.78 (m, 2H), 6.53 (d, *J* = 7.8 Hz, 1H), 5.70 (s, 1H), 5.58 (d, *J* = 15.0 Hz, 1H), 5.36 (s, 1H), 5.23 (d, *J* = 15.0 Hz, 1H), 5.09 (s, 1H), 3.70 (dd, *J* = 27.4, 13.5 Hz, 2H), 3.51 (d, *J* = 13.5 Hz, 1H), 3.28 – 3.20 (m, 1H). $^{13}$C NMR (101 MHz, DMSO-*d*$_6$) δ 155.9, 137.7, 136.7, 135.0, 132.7, 130.9, 129.5, 129.2, 128.9, 128.8, 128.7, 128.6, 128.4, 128.2, 127.9, 124.2, 111.0, 57.5, 56.5, 51.4, 50.7. HRMS (ESI) calcd for $C_{25}H_{23}N_5$ ([M+Na]$^+$): 416.1851, found 416.1834.

*2-Benzyl-1-(1-benzyl-1H-tetrazol-5-yl)-6-methyl-4-methylene-1,2,3,4-tetrahydroisoquinoline (**8b**)*

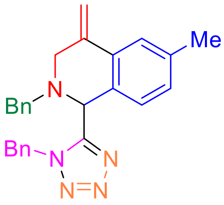


**8b**

Colorless oil, 288 mg, 71% yield. $^{1}$H NMR (300 MHz, DMSO-d$_6$) δ 7.71 – 7.63 (m, 1H), 7.31 (d, J = 2.2 Hz, 2H), 7.30 – 7.28 (m, 2H), 7.27 (d, J = 1.9 Hz, 2H), 7.14 (dd, J = 7.0, 2.5 Hz, 2H), 7.07 – 6.95 (m, 3H), 6.63 (d, J = 7.9 Hz, 1H), 5.82 (s, 1H), 5.78 – 5.70 (m, 1H), 5.51 (t, J = 7.7 Hz, 2H), 4.99 (s, 1H), 3.74 – 3.49 (m, 3H), 3.17 (d, J = 14.5 Hz, 1H), 2.31 (s, 3H). $^{13}$C NMR (75 MHz, DMSO-d$_6$) δ 156.0, 137.7, 137.6, 136.9, 135.0, 132.4, 129.8, 129.5, 129.1, 129.0, 128.8, 128.6, 128.5, 128.2, 128.1, 127.9, 124.4, 110.7, 57.6, 56.4, 51.6, 50.7, 21.3. HRMS (ESI) calcd for $C_{26}H_{25}N_5$ ([M+Na]$^+$): 430.2008, found 430.2052.

*2-Benzyl-1-(1-benzyl-1H-tetrazol-5-yl)-6-chloro-4-methylene-1,2,3,4-tetrahydroisoquinoline (**8c**)*

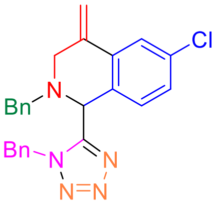


**8c**

Light yellow solid, 281 mg, 66 % yield. $^{1}$H NMR (400 MHz, Chloroform-*d*) δ 7.93 (d, *J* = 2.2 Hz, 1H), 7.35 – 7.31 (m, 3H), 7.26 (dt, *J* = 5.9, 2.5 Hz, 4H), 7.16 – 7.10 (m, 2H), 7.08 (dd, *J* = 4.5, 1.8 Hz, 2H), 6.89 (d, *J* = 8.3 Hz, 1H), 5.97 (s, 1H), 5.74 (d, *J* = 15.5 Hz, 1H), 5.56 (d, *J* = 15.6 Hz, 2H), 5.08 (s, 1H), 3.68 (d, *J* = 13.0 Hz, 1H), 3.57 (d, *J* = 13.1 Hz, 1H), 3.50 (d, *J* = 14.7 Hz, 1H), 3.19 (d, *J* = 14.9 Hz, 1H). $^{13}$C NMR (101 MHz, Chloroform-*d*) δ 160.4, 142.4, 140.0, 139.8, 139.5, 138.1, 135.7, 134.3, 133.9, 133.6, 133.4, 133.3, 132.8, 132.7, 128.6, 117.8, 62.0, 60.7, 55.4. HRMS (ESI) calcd for $C_{25}H_{22}ClN_5$ ([M+Na]$^+$): 450.1461, found 450.1505.

*2-Benzyl-1-(1-benzyl-1H-tetrazol-5-yl)-4-methylene-6-(trifluoromethyl)-1,2,3,4-tetrahydroisoquinoline (**8d**)*

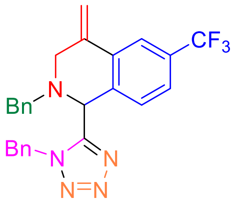

**8d**

Light yellow solid, 331 mg, 72% yield.$^1$H NMR (400 MHz, DMSO-$d_6$) δ 8.19 (d, $J$ = 1.9 Hz, 1H), 7.55 (dd, $J$ = 8.2, 1.9 Hz, 1H), 7.34 – 7.30 (m, 3H), 7.26 (d, $J$ = 2.5 Hz, 3H), 7.18 – 7.11 (m, 3H), 7.11 – 7.05 (m, 2H), 6.07 (d, $J$ = 1.3 Hz, 1H), 5.77 (d, $J$ = 15.5 Hz, 1H), 5.71 (d, $J$ = 3.6 Hz, 1H), 5.60 (d, $J$ = 15.5 Hz, 1H), 5.15 (s, 1H), 3.71 (d, $J$ = 13.1 Hz, 1H), 3.64 – 3.50 (m, 2H), 3.24 (d, $J$ = 15.0 Hz, 1H). $^{13}$C NMR (101 MHz, DMSO-$d_6$) δ 155.5, 137.6, 135.2, 134.0, 134.9, 133.8, 130.3, 129.5, 129.5, 129.3, 129.1, 129.1, 129.1, 129.0, 128.8, 128.6, 128.6, 128.1, 128.0, 127.9, 125.9, 125.0, 124.9, 123.2, 121.1, 121.1, 113.6, 57.3, 56.2, 50.7. $^{19}$F NMR (376 MHz, DMSO-$d_6$) δ -61.1. HRMS (ESI) calcd for $C_{26}H_{22}F_3N_5$ ([M+Na]$^+$): 484.1725, found 484.1752.

*2-Benzyl-1-(1-benzyl-1H-tetrazol-5-yl)-7-methoxy-4-methylene-1,2,3,4-tetrahydroisoquinoline (**8e**)*

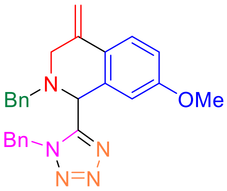

**8e**

Light yellow solid, 287 mg, 68% yield. $^1$H NMR (400 MHz, DMSO-$d_6$) δ 7.80 (d, $J$ = 8.8 Hz, 1H), 7.34 – 7.30 (m, 3H), 7.28 (d, $J$ = 2.0 Hz, 1H), 7.27 (d, $J$ = 1.9 Hz, 2H), 7.19 – 7.10 (m, 2H), 7.10 – 7.01 (m, 2H), 6.91 (dd, $J$ = 8.8, 2.7 Hz, 1H), 6.28 (d, $J$ = 2.6 Hz, 1H), 5.74 (s, 3H), 5.68 (s, 1H), 5.55 – 5.48 (m, 2H), 4.87 (s, 1H), 3.66 (d, $J$ = 13.1 Hz, 1H), 3.61 (s, 3H), 3.17 (d, $J$ = 14.7 Hz, 1H). $^{13}$C NMR (101 MHz, DMSO-$d_6$) δ 159.7, 155.8, 137.8, 136.3, 135.1, 132.2, 129.5, 129.1, 128.8, 128.6, 128.1, 127.9, 125.8, 125.5, 115.1, 112.7, 108.6, 57.5, 56.5, 55.6, 55.3, 51.5, 50.7. HRMS (ESI) calcd for $C_{26}H_{25}N_5O$ ([M+Na]$^+$): 446.1957, found 446.1961.

*6-Benzyl-5-(1-benzyl-1H-tetrazol-5-yl)-8-methylene-5,6,7,8-tetrahydro-[1,3]dioxolo[4,5-g]isoquinoline (**8f**)*

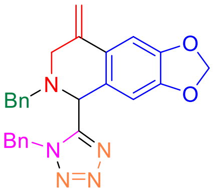

**8f**

Light yellow solid, 319 mg, 73% yield.$^1$H NMR (400 MHz, DMSO-$d_6$) δ 7.39 (s, 1H), 7.36 – 7.30 (m, 3H), 7.27 (dd, $J$ = 5.0, 1.9 Hz, 3H), 7.17 – 7.10 (m, 2H), 7.09 – 7.02 (m, 2H), 6.27 (s, 1H), 6.02 (d, $J$ = 1.0 Hz, 1H), 5.98 (d, $J$ = 1.0 Hz, 1H), 5.74 – 5.68 (m, 2H), 5.52 (d, $J$ = 15.4 Hz, 1H), 5.42 (s, 1H), 4.89 (s, 1H), 3.74 – 3.55 (m, 2H), 3.54 – 3.45 (m, 1H), 3.14 (d, $J$ = 14.7 Hz, 1H). $^{13}$C NMR (101 MHz, DMSO-$d_6$) δ 155.9, 148.1, 148.0, 137.8, 136.4, 135.1, 129.5, 129.1, 128.8, 128.6, 128.1, 127.9, 127.0, 124.7, 109.7, 107.8, 103.7, 101.7, 57.4, 56.5, 51.3, 50.6. HRMS (ESI) calcd for $C_{26}H_{23}N_5O_2$ ([M+Na]$^+$): 460.1749, found 460.1803.

*2-Methyl-4-methylene-1-(1-(2,4,4-trimethylpentan-2-yl)-1H-tetrazol-5-yl)-1,2,3,4-tetrahydroisoquinoline (**8g**)*

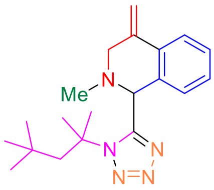

**8g**

White solid, 230 mg, 68% yield. $^1$H NMR (400 MHz, Chloroform-$d$) δ 7.78 (d, $J$ = 7.9 Hz, 1H), 7.31 (t, $J$ = 7.6 Hz, 1H), 7.22 (t, $J$ = 7.5 Hz, 1H), 6.81 (d, $J$ = 7.7 Hz, 1H), 5.76 (s, 1H), 5.33 (s, 1H), 5.12 (s, 1H), 3.85 (d, $J$ = 14.4 Hz, 1H), 3.23 (d, $J$ = 14.4 Hz, 1H), 2.43 (s, 3H), 2.11 (d, $J$ = 14.7 Hz, 2H), 1.81 (s, 6H), 0.85 (s, 9H). $^{13}$C NMR (101 MHz, Chloroform-$d$) δ 155.0, 132.4, 128.4, 127.8, 123.8, 110.0, 53.3, 31.6, 30.8, 29.8. HRMS (ESI) calcd for $C_{20}H_{29}N_5$ ([M+Na]$^+$): 362.2321, found 362.2314.

*6-Chloro-2-methyl-4-methylene-1-(1-(2,4,4-trimethylpentan-2-yl)-1H-tetrazol-5-yl)-1,2,3,4-tetrahydroisoquinoline(**8h**)*

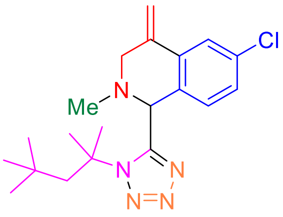

**8h**

Light yellow solid, 257 mg, 69% yield.$^1$H NMR (400 MHz, Chloroform-$d$) δ 7.71 (d, $J$ = 2.2 Hz, 1H), 7.16 (dd, $J$ = 8.2, 2.1 Hz, 1H), 6.75 (d, $J$ = 8.3 Hz, 1H), 5.72 (s, 1H), 5.23 (s, 1H), 5.13 (s, 1H), 3.77 (d, $J$ = 14.8 Hz, 1H), 3.18 (d, $J$ = 14.7 Hz, 1H), 2.40 (s, 3H), 2.19 (d, $J$ = 15.3 Hz, 1H), 2.05 (d, $J$ = 14.9 Hz, 1H), 1.80 (s, 6H), 0.82 (s, 9H). $^{13}$C NMR (101 MHz, Chloroform-$d$) δ 154.5, 134.2, 133.9, 129.6, 128.5, 123.8, 111.6, 66.3, 53.3, 31.6, 30.8, 30.1, 29.7. HRMS (ESI) calcd for $C_{20}H_{28}ClN_5$ ([M+Na]$^+$): 396.1931, found 396.1923.

*6,7-Dimethoxy-2-methyl-4-methylene-1-(1-(2,4,4-trimethylpentan-2-yl)-1H-tetrazol-5-yl)-1,2,3,4-tetrahydroisoquinoline (**8i**)*

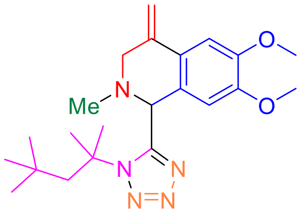


**8i**

Light yellow solid, 263 mg, 66% yield. $^{1}$H NMR (400 MHz, Chloroform-*d*) δ 7.16 (s, 1H), 6.18 (s, 1H), 5.54 (s, 1H), 5.22 (s, 1H), 4.99 (s, 1H), 3.90 (s, 3H), 3.74 (dd, *J* = 14.3, 2.7 Hz, 1H), 3.69 (s, 3H), 3.16 (d, *J* = 14.3 Hz, 1H), 2.38 (s, 3H), 2.13 (d, *J* = 10.3 Hz, 2H), 1.76 (d, *J* = 8.8 Hz, 6H), 0.82 (s, 9H).

$^{13}$C NMR (101 MHz, Chloroform-*d*) δ 155.07, 149.6, 148.7, 125.2, 108.0, 105.9, 55.9, 55.8, 53.3, 31.6, 30.9, 30.1. HRMS (ESI) calcd for $C_{22}H_{33}N_5O_2$ ([M+Na]$^+$): 422.2532, found 422.2106

*6-Methyl-8-methylene-5-(1-(2,4,4-trimethylpentan-2-yl)-1H-tetrazol-5-yl)-5,6,7,8-tetrahydro-[1,3]dioxolo[4,5-g]isoquinoline (**8j**)*

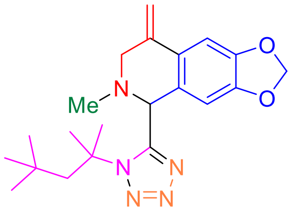


**8j**

White solid, 252 mg, 66% yield. $^{1}$H NMR (400 MHz, Chloroform-*d*) δ 7.18 (s, 1H), 6.22 (s, 1H), 5.94 (d, *J* = 1.2 Hz, 1H), 5.91 (d, *J* = 1.3 Hz, 1H), 5.53 (s, 1H), 5.15 (s, 1H), 4.98 (s, 1H), 3.75 (d, *J* = 14.6 Hz, 1H), 3.13 (d, *J* = 14.6 Hz, 1H), 2.41 (s, 3H), 2.20 (d, *J* = 15.4 Hz, 1H), 2.06 (s, 1H), 1.83 (d, *J* = 13.0 Hz, 6H), 0.84 (s, 9H). $^{13}$C NMR (101 MHz, Chloroform-*d*) δ 154.9, 148.1, 147.7, 126.7, 108.6, 107.3, 103.4, 101.2, 53.2, 31.6, 30.8, 29.6. HRMS (ESI) calcd for $C_{21}H_{29}N_5O_2$ ([M+Na]$^+$): 406.2219, found 406.2231.

## 3. X-ray report of 6d and 8c

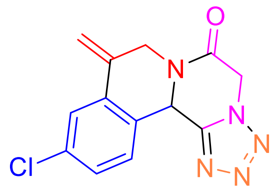


**6d**

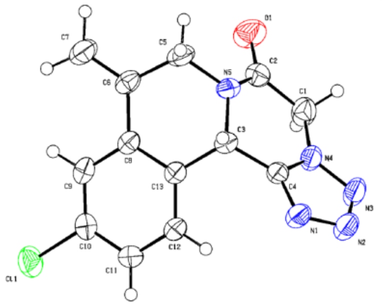

**CCDC: 2164364**

Bond precision: C-C = 0.0020 A Wavelength=0.71073

| | | | |
|---|---|---|---|
| Cell: | a=8.5083(11) | b=14.3480(17) | c=10.4672(13) |
| | alpha=90 | beta=105.064(2) | gamma=90 |
| Temperature: | 296 K | | |

| | Calculated | Reported |
|---|---|---|
| Volume | 1233.9(3) | 1233.9(3) |
| Space group | P 21/c | P 21/c |
| Hall group | -P 2ybc | -P 2ybc |
| Moiety formula | C13 H10 Cl N5 O | ? |
| Sum formula | C13 H10 Cl N5 O | C13 H10 Cl N5 O |
| Mr | 287.71 | 287.71 |
| Dx,g cm-3 | 1.549 | 1.549 |
| Z | 4 | 4 |
| Mu (mm-1) | 0.313 | 0.313 |
| F000 | 592.0 | 592.0 |
| F000' | 592.78 | |
| h,k,lmax | 10,17,12 | 10,17,12 |
| Nref | 2165 | 2159 |
| Tmin,Tmax | 0.922,0.928 | |
| Tmin' | 0.922 | |

Correction method= Not given

Data completeness= 0.997 Theta(max)= 24.993

R(reflections)= 0.0316( 1864) wR2(reflections)= 0.0829( 2159)

S = 1.086 Npar= 190

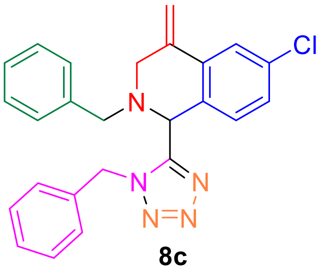


8c

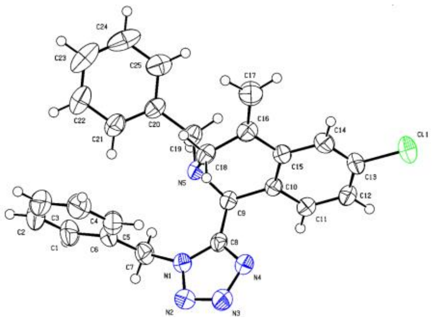

**CCDC: 2321622**

---

| | | | |
|---|---|---|---|
| Bond precision: | C-C = 0.0034 A | | Wavelength=0.71073 |
| Cell: | a=21.3467(15) | b=14.9952(11) | c=6.8948(5) |
| | alpha=90 | beta=90.794(1) | gamma=90 |
| Temperature: | 296 K | | |

| | Calculated | Reported |
|---|---|---|
| Volume | 2206.8(3) | 2206.8(3) |
| Space group | P 21/c | P 21/c |
| Hall group | -P 2ybc | -P 2ybc |
| Moiety formula | C25 H22 Cl N5 | ? |
| Sum formula | C25 H22 Cl N5 | C25 H22 Cl N5 |
| Mr | 427.93 | 427.92 |
| Dx,g cm-3 | 1.288 | 1.288 |
| Z | 4 | 4 |
| Mu (mm-1) | 0.195 | 0.195 |
| F000 | 896.0 | 896.0 |
| F000’ | 896.86 | |
| h,k,lmax | 25,17,8 | 25,17,8 |
| Nref | 3888 | 3882 |
| Tmin,Tmax | 0.954,0.973 | |
| Tmin’ | 0.951 | |

Correction method= Not given

Data completeness= 0.998 Theta(max)= 24.999

R(reflections)= 0.0424( 2407) wR2(reflections)= 0.1147( 3882)

S = 0.862 Npar= 288

## 4. $^{1}$H NMR, $^{13}$C NMR of products 5a, 6 and 8

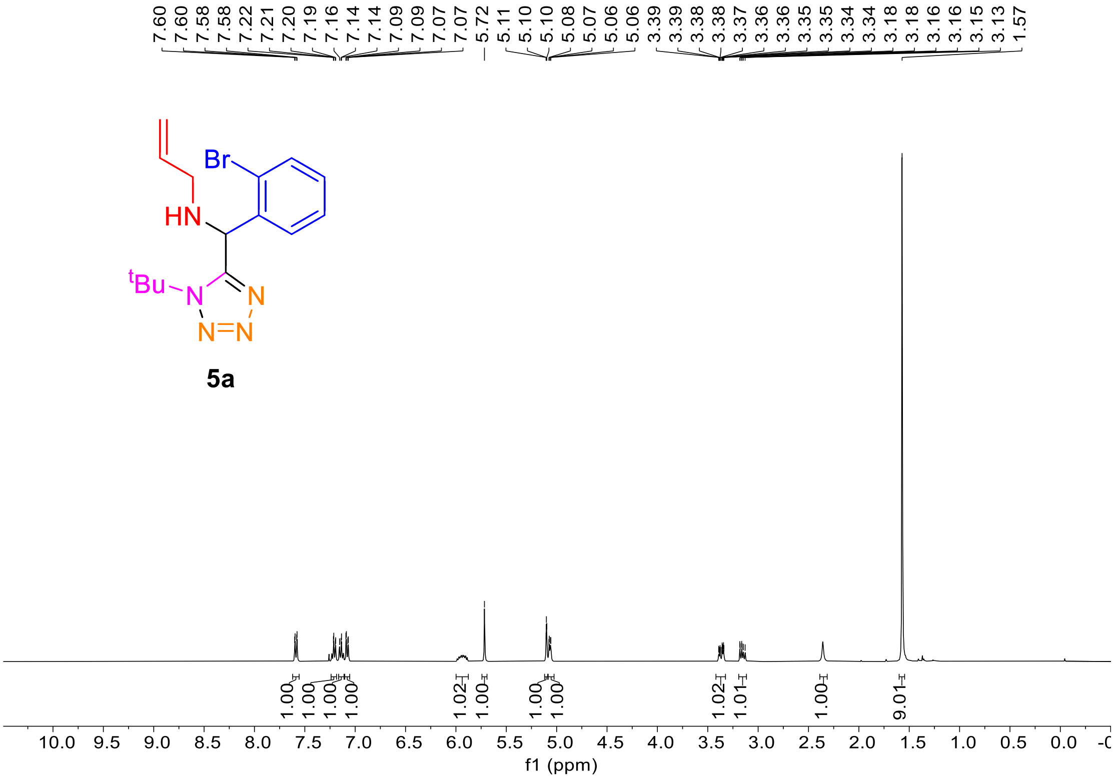


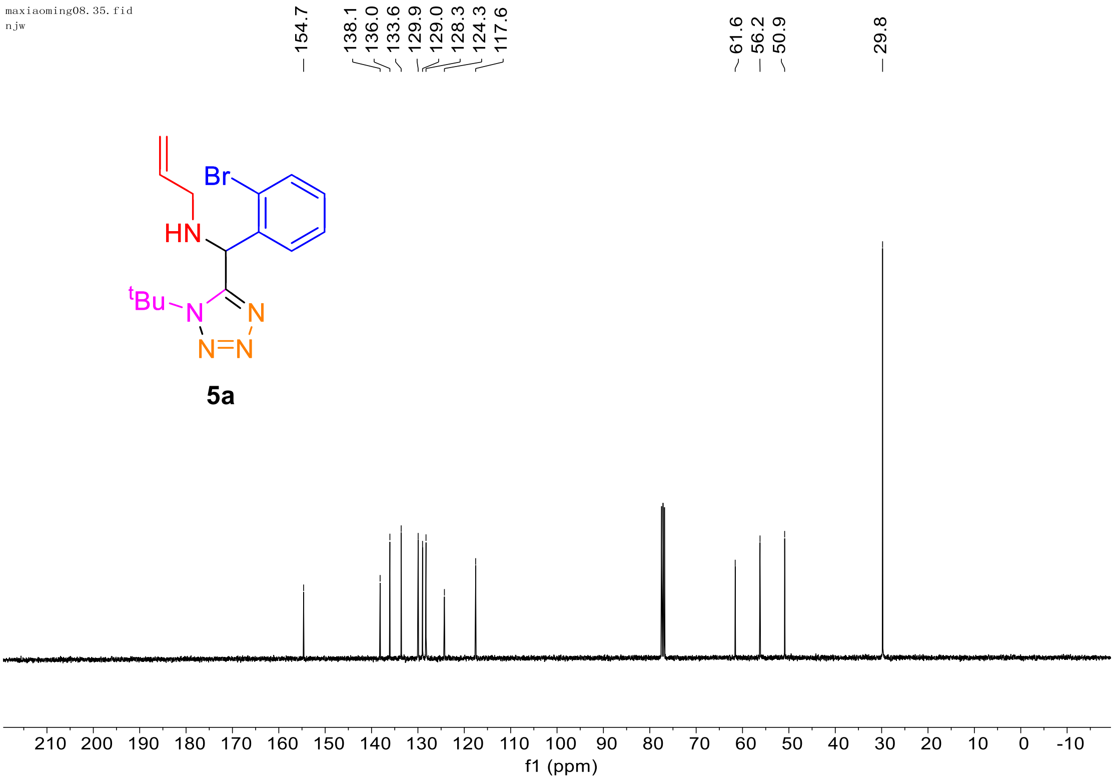

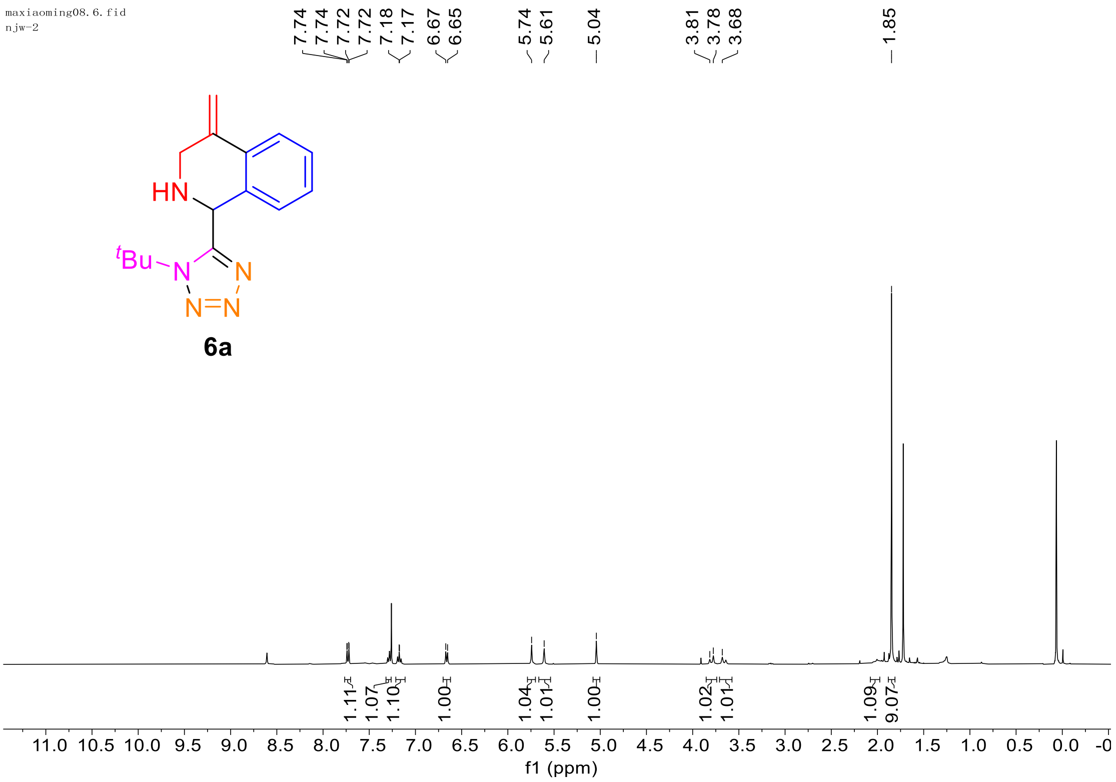
maxiaoming08.6.fid
njw-2
7.74
7.74
7.72
7.72
7.18
7.17
6.67
6.65
5.74
5.61
5.04
3.81
3.78
3.68
1.85
HN
tBu
N
N
N=N
6a
1.11
1.07
1.10
1.00
1.04
1.01
1.00
1.02
1.01
1.09
9.07
11.0 10.5 10.0 9.5 9.0 8.5 8.0 7.5 7.0 6.5 6.0 5.5 5.0 4.5 4.0 3.5 3.0 2.5 2.0 1.5 1.0 0.5 0.0
f1 (ppm)

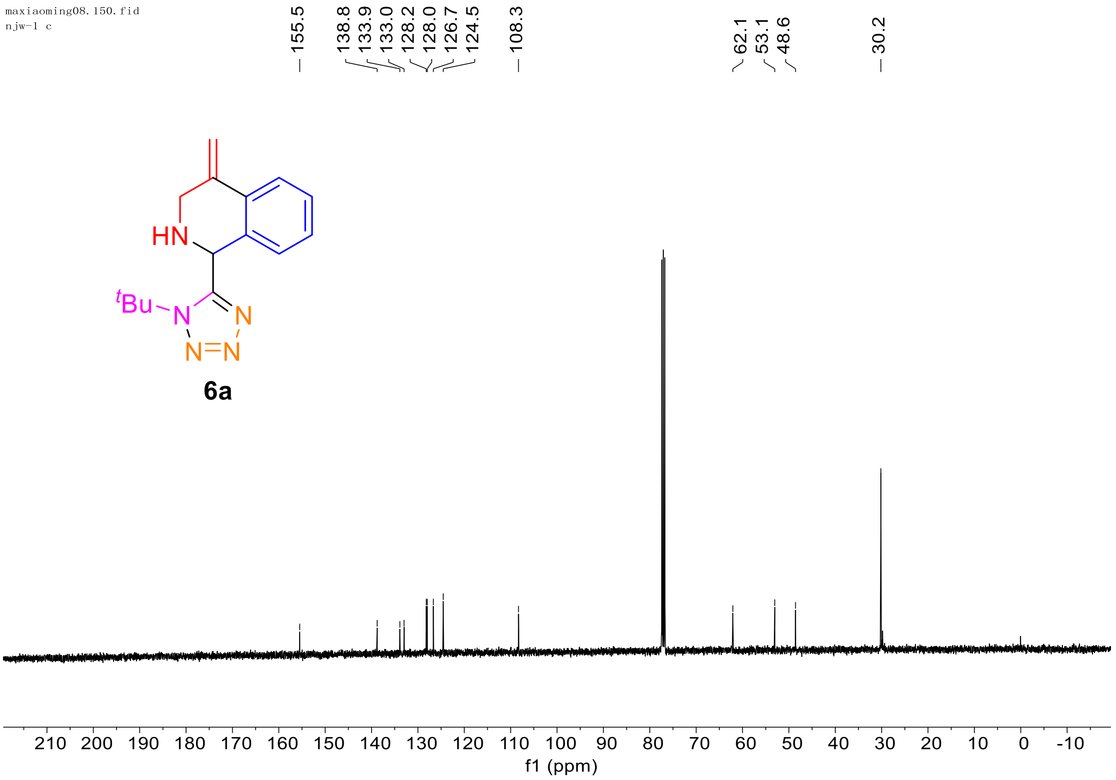
maxiaoming08.150.fid
njw-1 c
155.5
138.8
133.9
133.0
128.2
128.0
126.7
124.5
108.3
62.1
53.1
48.6
30.2
HN
tBu
N
N
N=N
6a
210 200 190 180 170 160 150 140 130 120 110 100 90 80 70 60 50 40 30 20 10 0 -10
f1 (ppm)

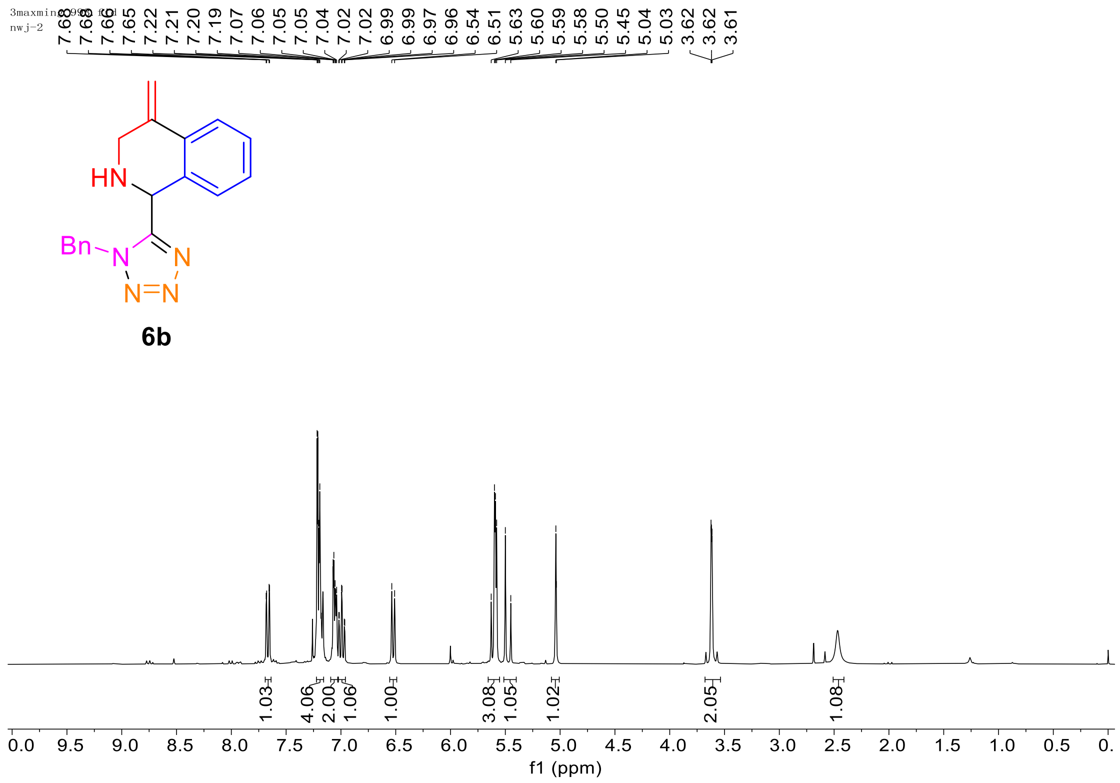
nwj-2
7.68 7.68 7.66 7.65 7.22 7.21 7.20 7.19 7.07 7.06 7.05 7.05 7.04 7.02 7.02 6.99 6.99 6.97 6.96 6.54 6.51 5.63 5.60 5.59 5.58 5.50 5.45 5.04 5.03 3.62 3.62 3.61
HN
Bn
N
N=N
6b
1.03 4.06 2.00 1.06 1.00 3.08 1.05 1.02 2.05 1.08
0.0 9.5 9.0 8.5 8.0 7.5 7.0 6.5 6.0 5.5 5.0 4.5 4.0 3.5 3.0 2.5 2.0 1.5 1.0 0.5 0.
f1 (ppm)

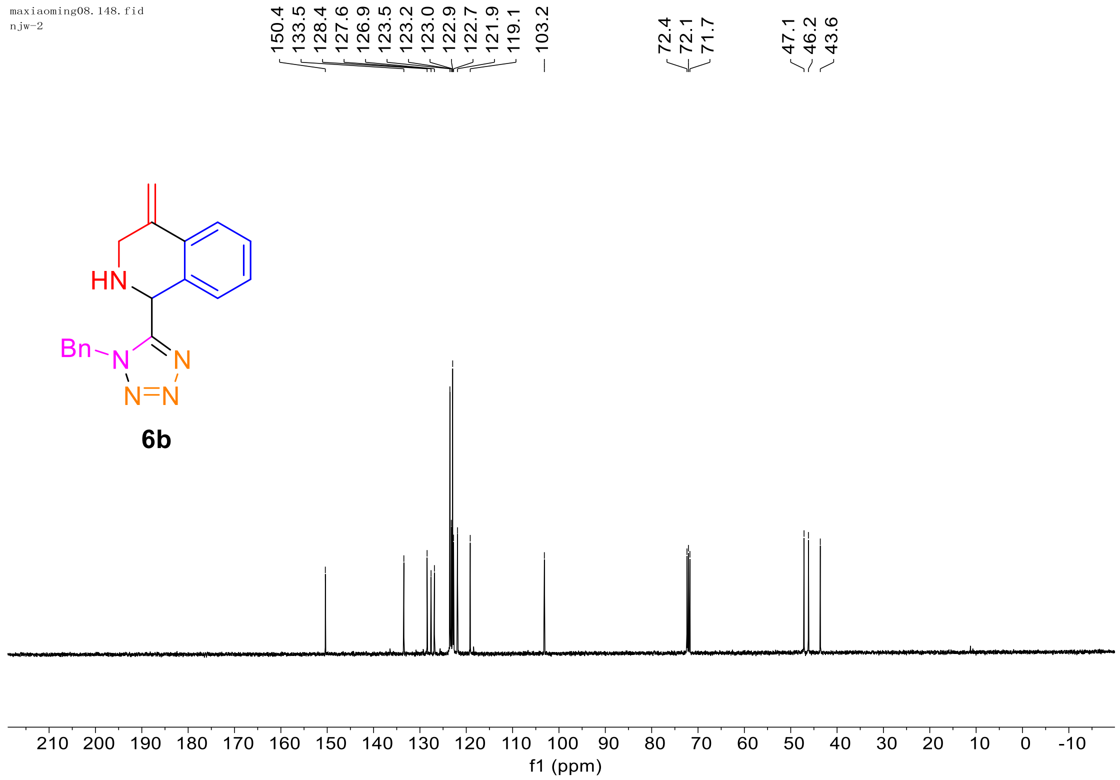
maxiaoming08.148.fid
njw-2
150.4 133.5 128.4 127.6 126.9 123.5 123.2 123.0 122.9 122.7 121.9 119.1 103.2
72.4 72.1 71.7
47.1 46.2 43.6
HN
Bn
N
N=N
6b
210 200 190 180 170 160 150 140 130 120 110 100 90 80 70 60 50 40 30 20 10 0 -10
f1 (ppm)

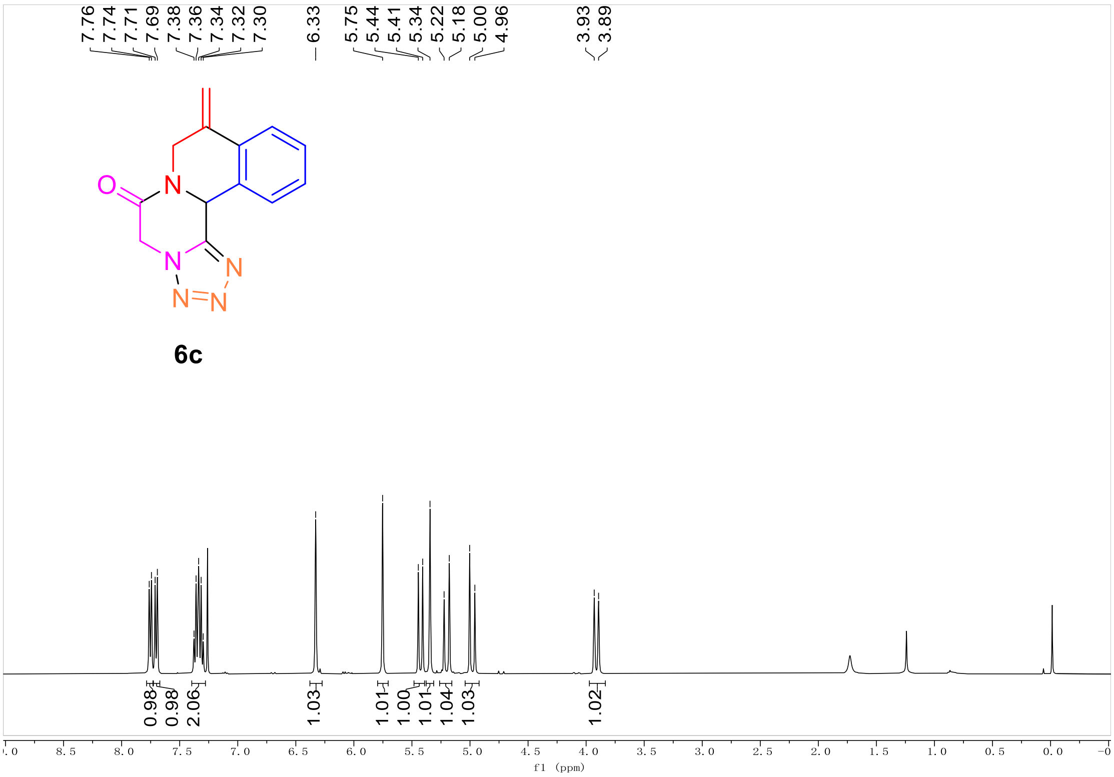
7.76
7.74
7.71
7.69
7.38
7.36
7.34
7.32
7.30
6.33
5.75
5.44
5.41
5.34
5.22
5.18
5.00
4.96
3.93
3.89
6c
0.98
0.98
2.06
1.03
1.01
1.00
1.01
1.04
1.03
1.02
f1 (ppm)

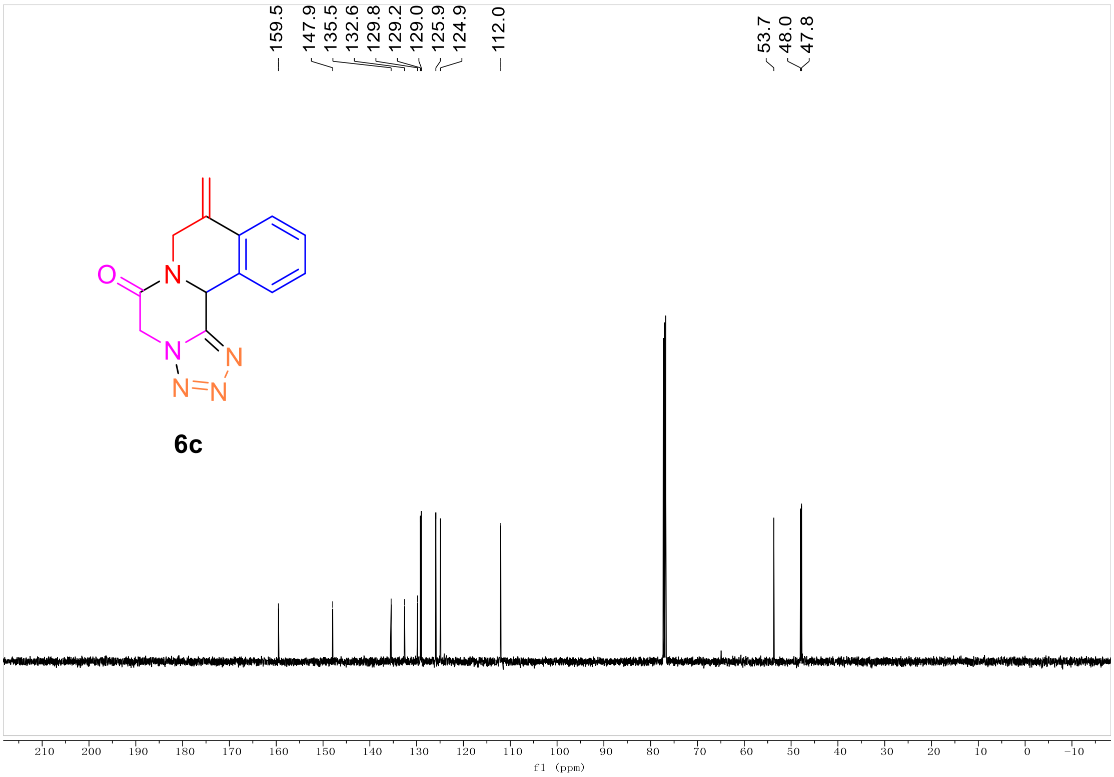
159.5
147.9
135.5
132.6
129.8
129.2
129.0
125.9
124.9
112.0
53.7
48.0
47.8
6c
f1 (ppm)

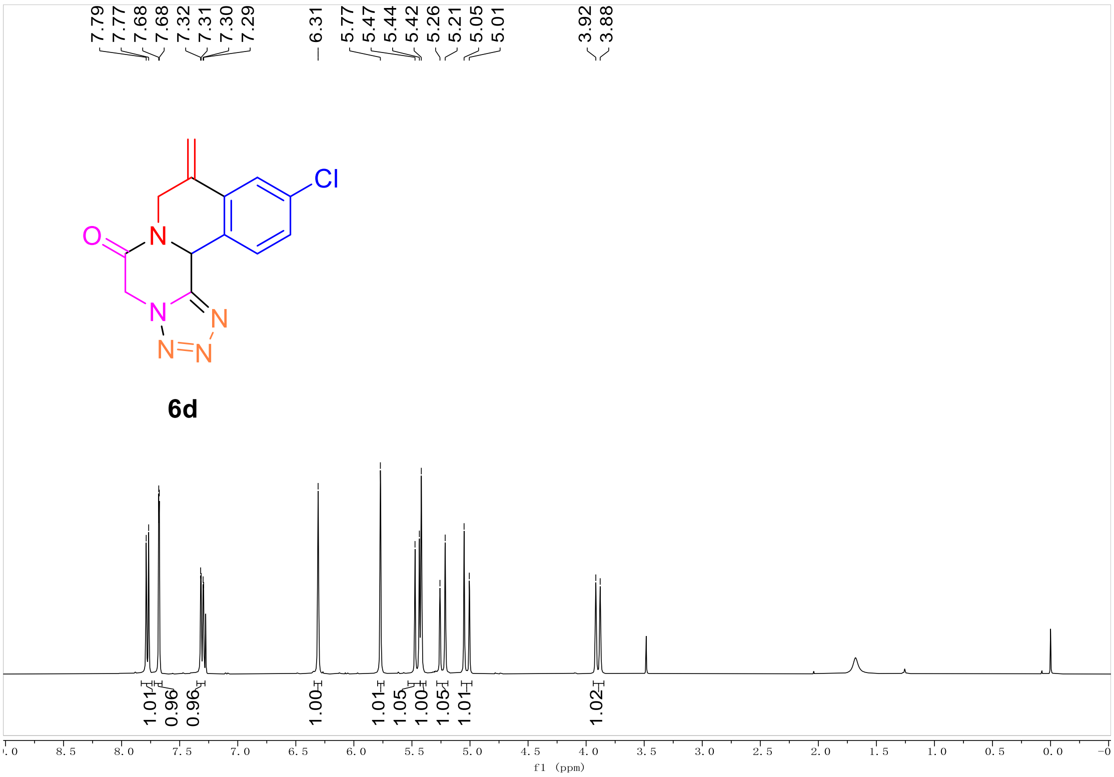
7.79
7.77
7.68
7.68
7.32
7.31
7.30
7.29
6.31
5.77
5.47
5.44
5.42
5.26
5.21
5.05
5.01
3.92
3.88
Cl
O
N
N
N
N=N
6d
1.01
0.96
0.96
1.00
1.01
1.05
1.00
1.05
1.01
1.02
f1 (ppm)

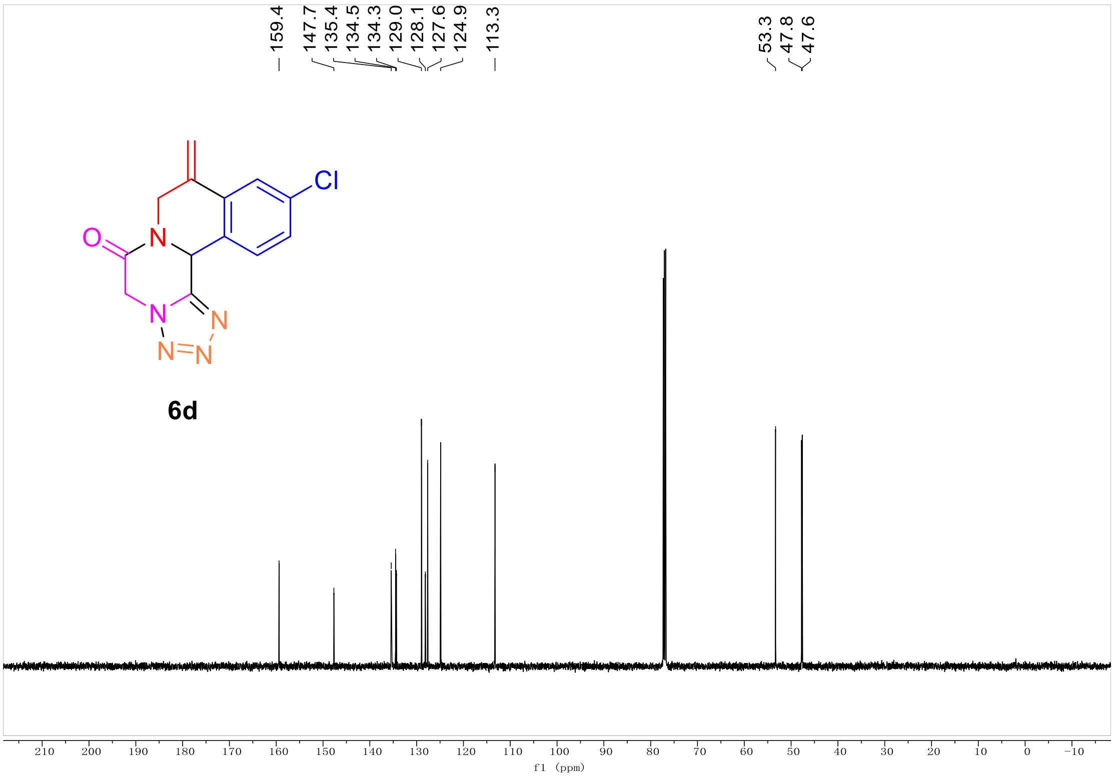
159.4
147.7
135.4
134.5
134.3
129.0
128.1
127.6
124.9
113.3
53.3
47.8
47.6
Cl
O
N
N
N
N=N
6d
f1 (ppm)

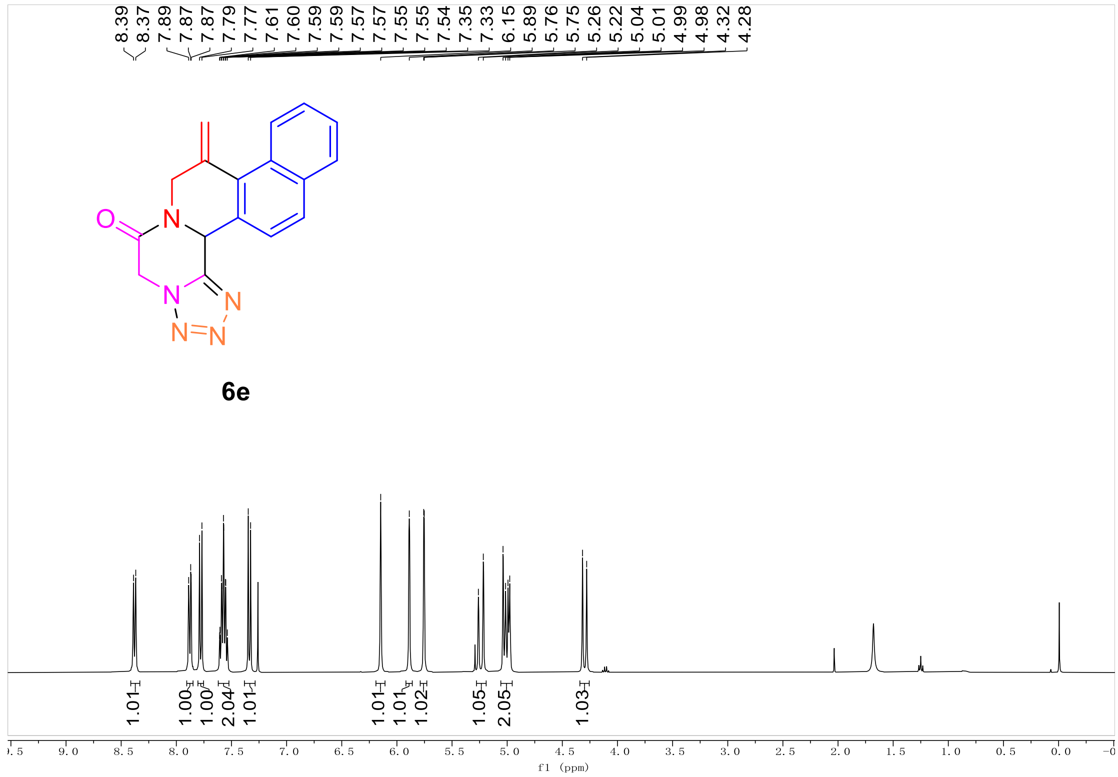
8.39
8.37
7.89
7.87
7.87
7.79
7.77
7.61
7.60
7.59
7.59
7.57
7.57
7.55
7.55
7.54
7.35
7.33
6.15
5.89
5.76
5.75
5.26
5.22
5.04
5.01
4.99
4.98
4.32
4.28
6e
1.01
1.00
1.00
2.04
1.01
1.01
1.01
1.02
1.05
2.05
1.03
f1 (ppm)

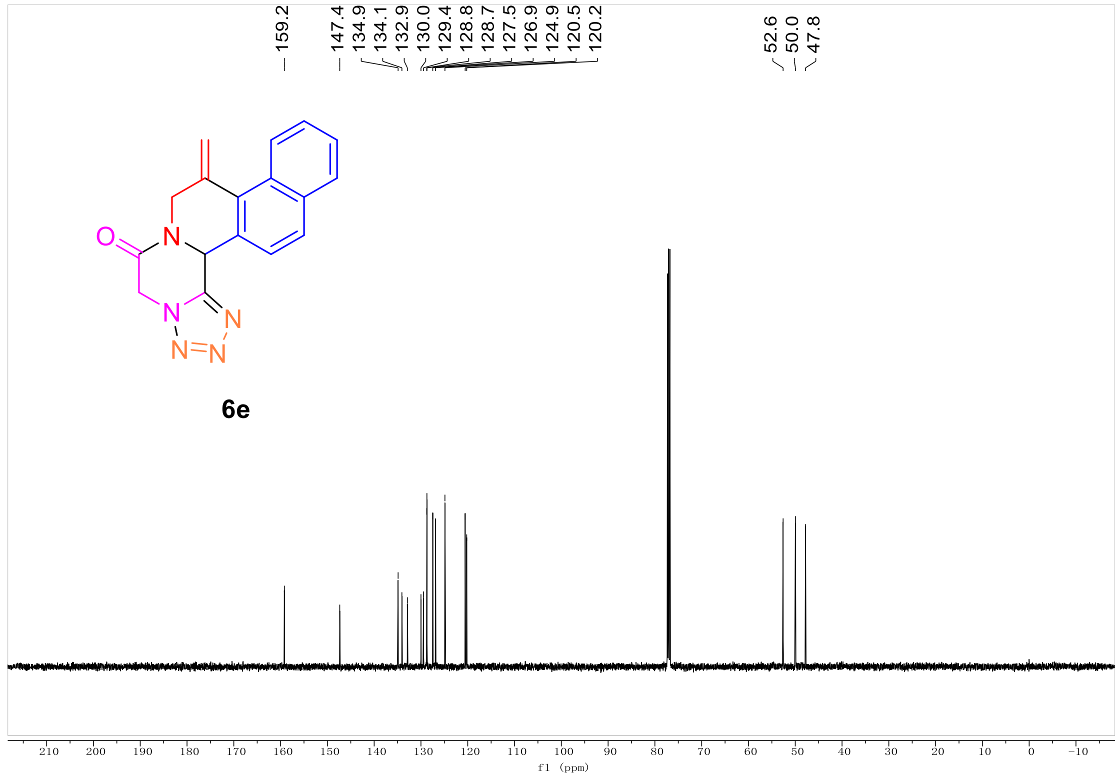
159.2
147.4
134.9
134.1
132.9
130.0
129.4
128.8
128.7
127.5
126.9
124.9
120.5
120.2
52.6
50.0
47.8
6e
f1 (ppm)

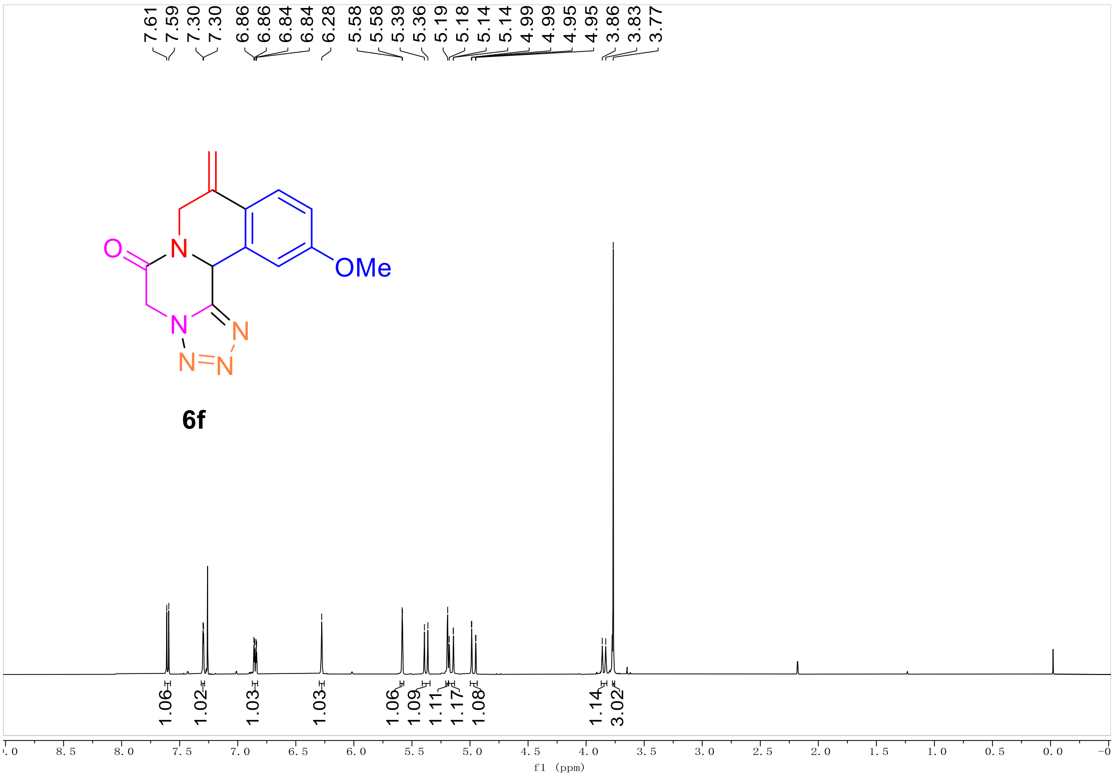
7.61
7.59
7.30
7.30
6.86
6.86
6.84
6.84
6.28
5.58
5.58
5.39
5.36
5.19
5.18
5.14
5.14
4.99
4.99
4.95
4.95
3.86
3.83
3.77
OMe
6f
1.06
1.02
1.03
1.03
1.06
1.09
1.11
1.17
1.08
1.14
3.02
f1 (ppm)

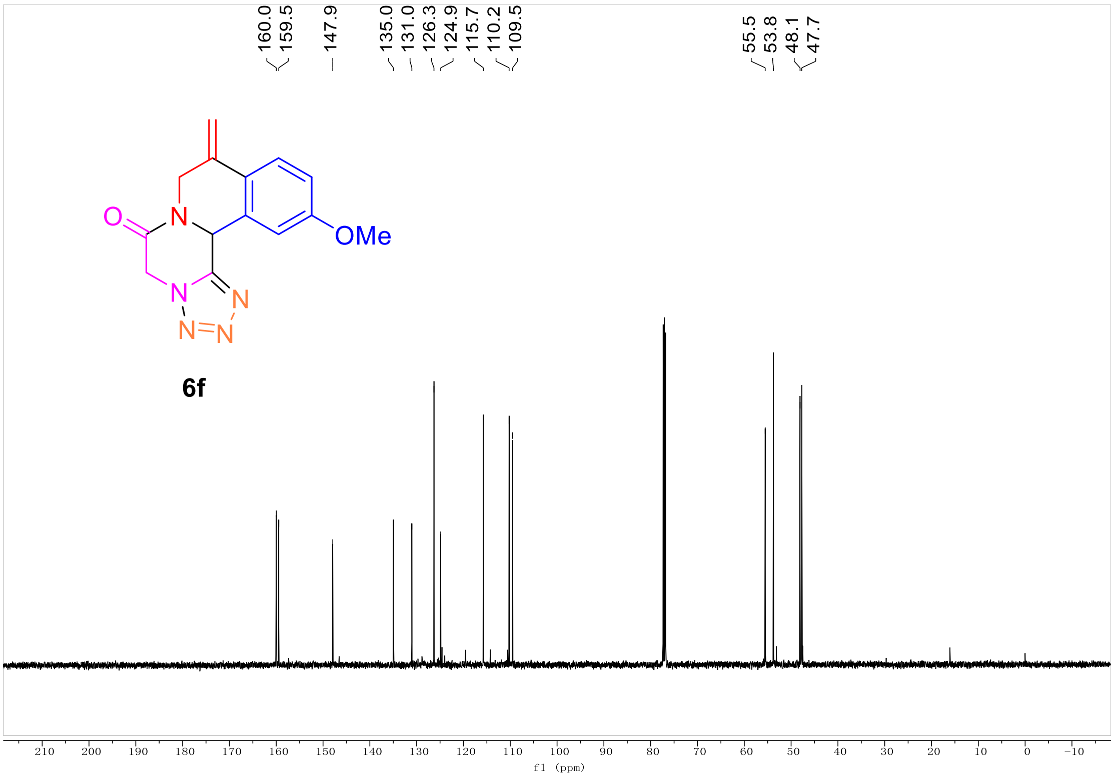
160.0
159.5
147.9
135.0
131.0
126.3
124.9
115.7
110.2
109.5
55.5
53.8
48.1
47.7
OMe
6f
f1 (ppm)

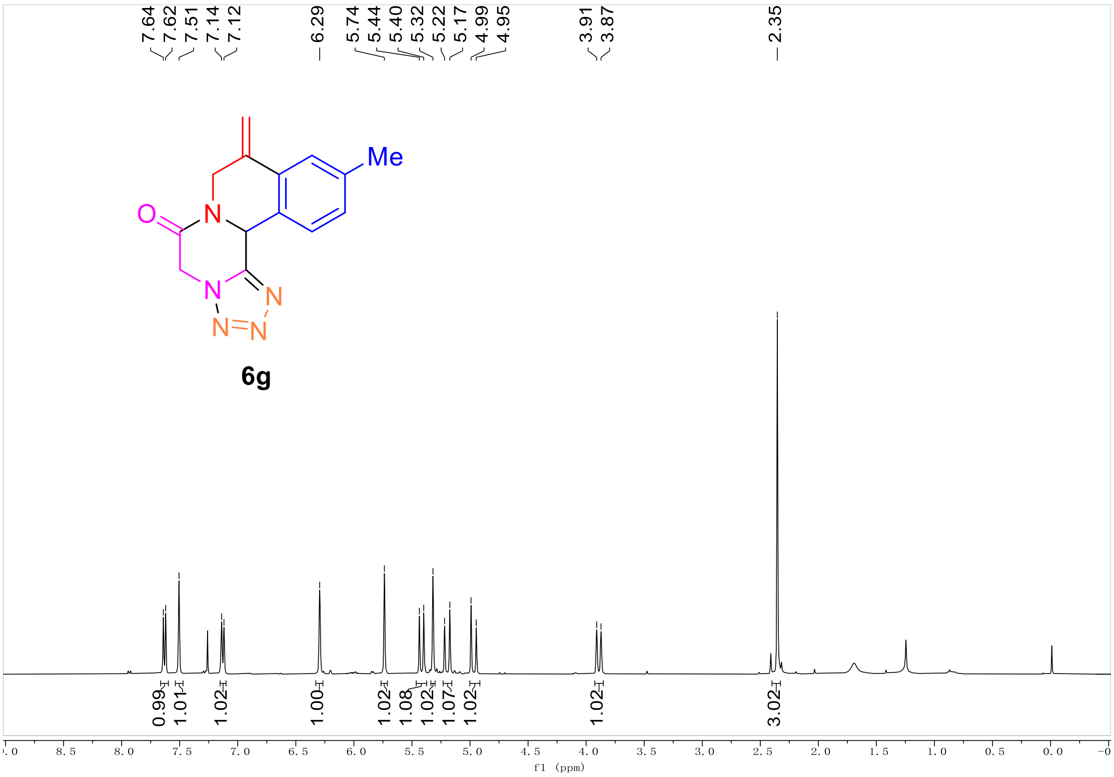

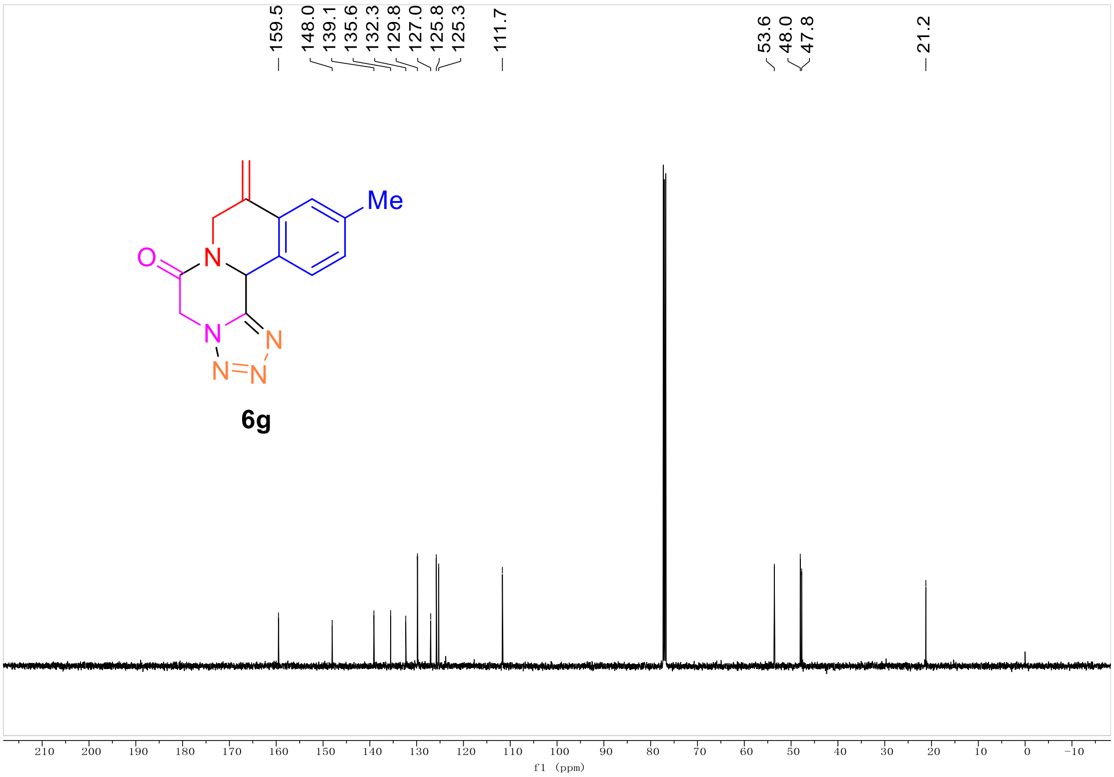

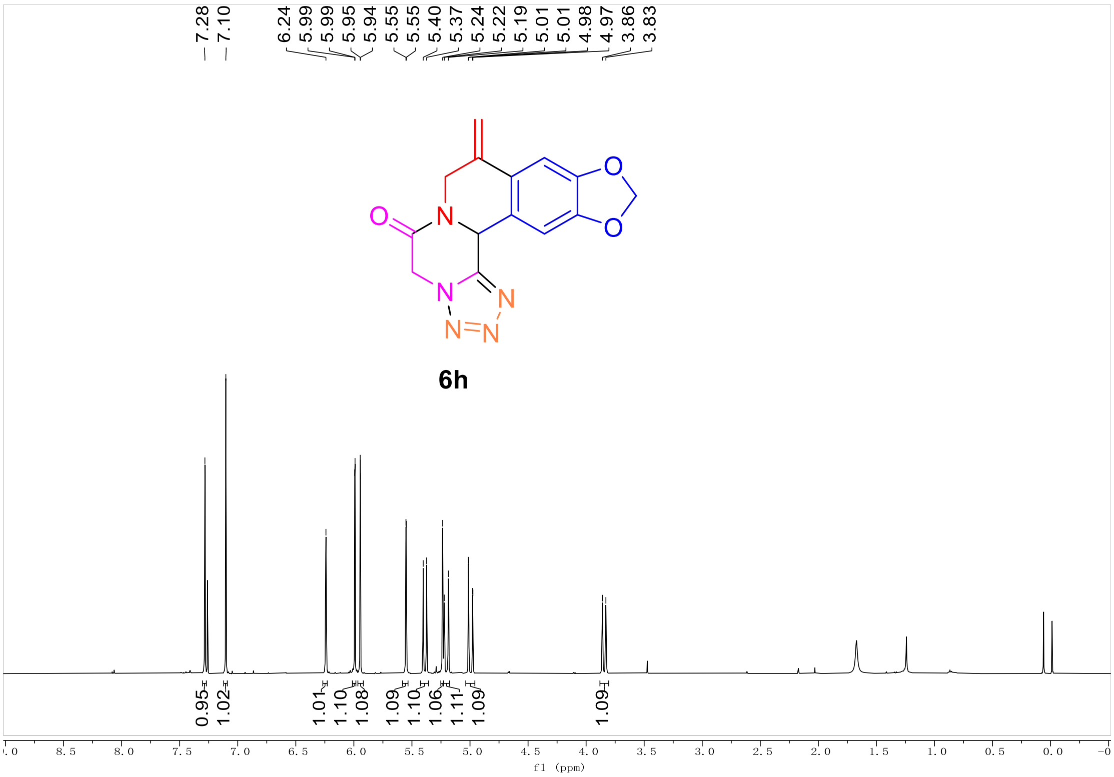
7.28
7.10
6.24
5.99
5.99
5.95
5.94
5.55
5.55
5.40
5.37
5.24
5.22
5.19
5.01
4.98
4.97
3.86
3.83
6h
0.95
1.02
1.01
1.10
1.08
1.09
1.10
1.06
1.11
1.09
1.09
f1 (ppm)

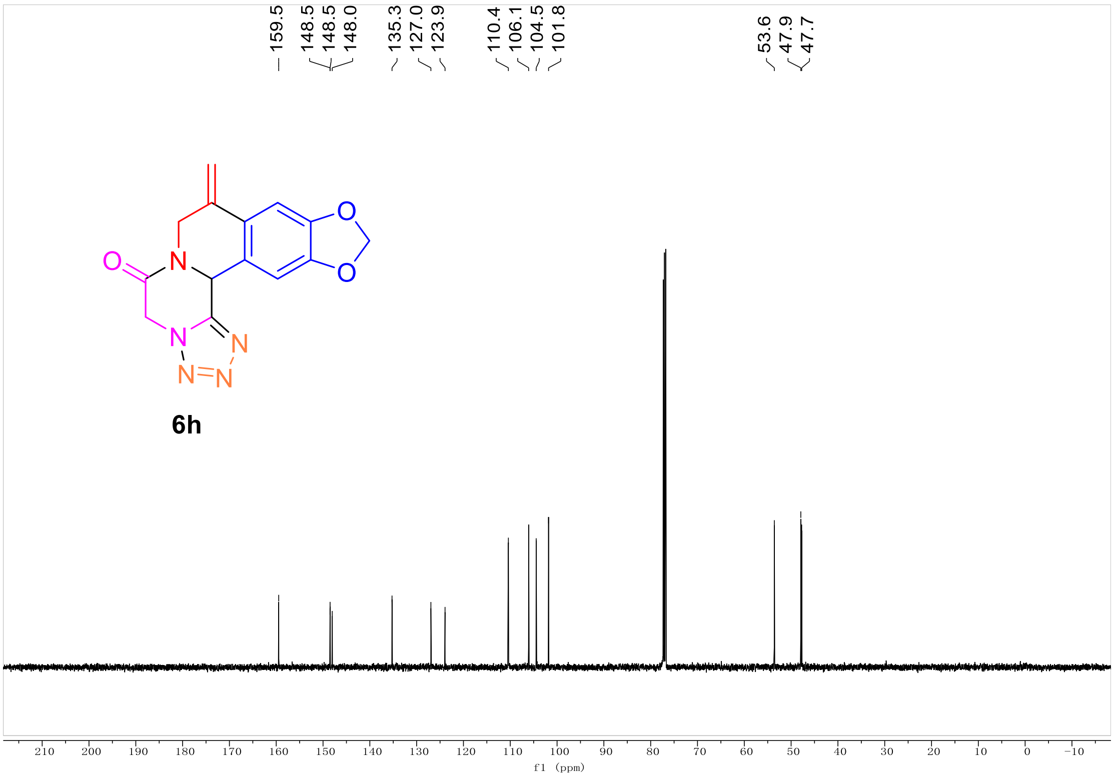
159.5
148.5
148.5
148.0
135.3
127.0
123.9
110.4
106.1
104.5
101.8
53.6
47.9
47.7
6h
f1 (ppm)

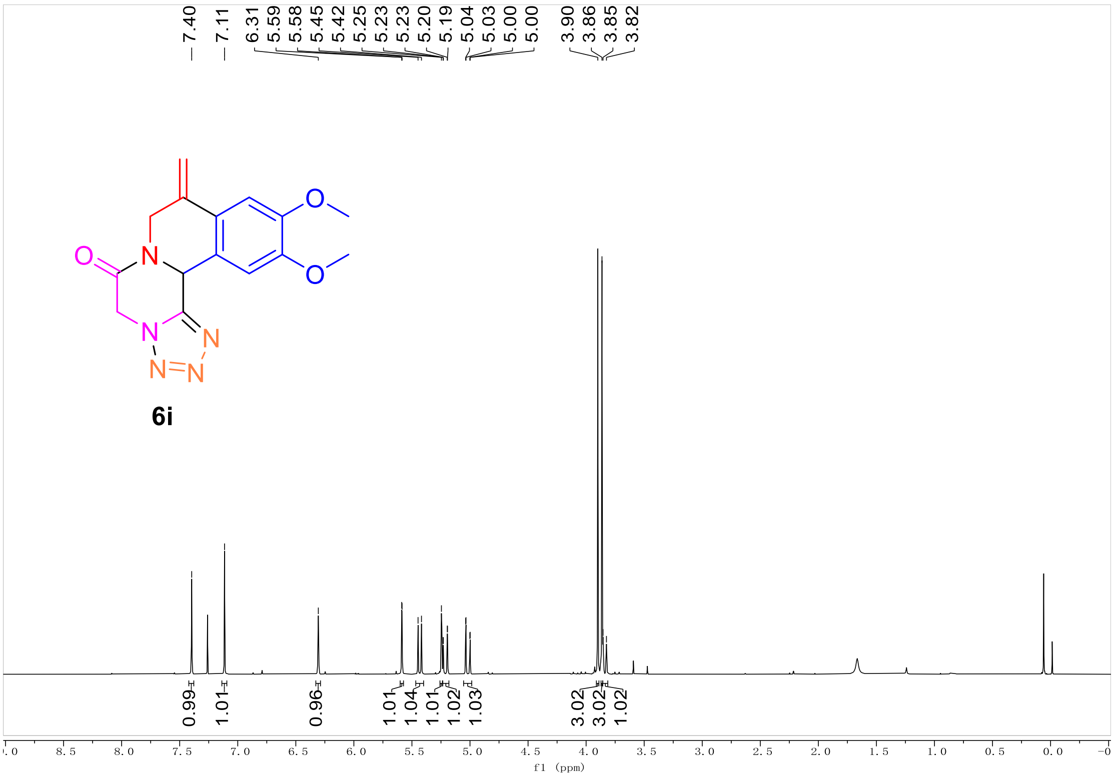
7.40
7.11
6.31
5.59
5.58
5.45
5.42
5.25
5.23
5.23
5.20
5.19
5.04
5.03
5.00
5.00
3.90
3.86
3.85
3.82
6i
0.99
1.01
0.96
1.01
1.04
1.01
1.02
1.03
3.02
3.02
1.02
f1 (ppm)

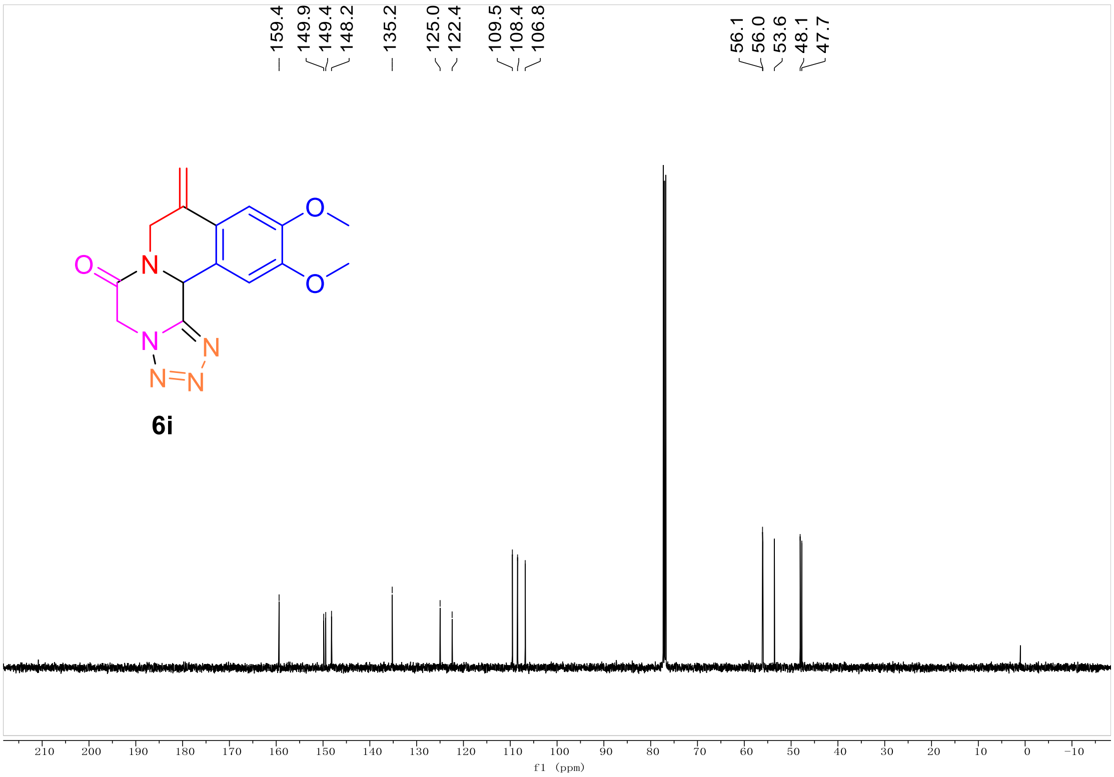
159.4
149.9
149.4
148.2
135.2
125.0
122.4
109.5
108.4
106.8
56.1
56.0
53.6
48.1
47.7
6i
f1 (ppm)

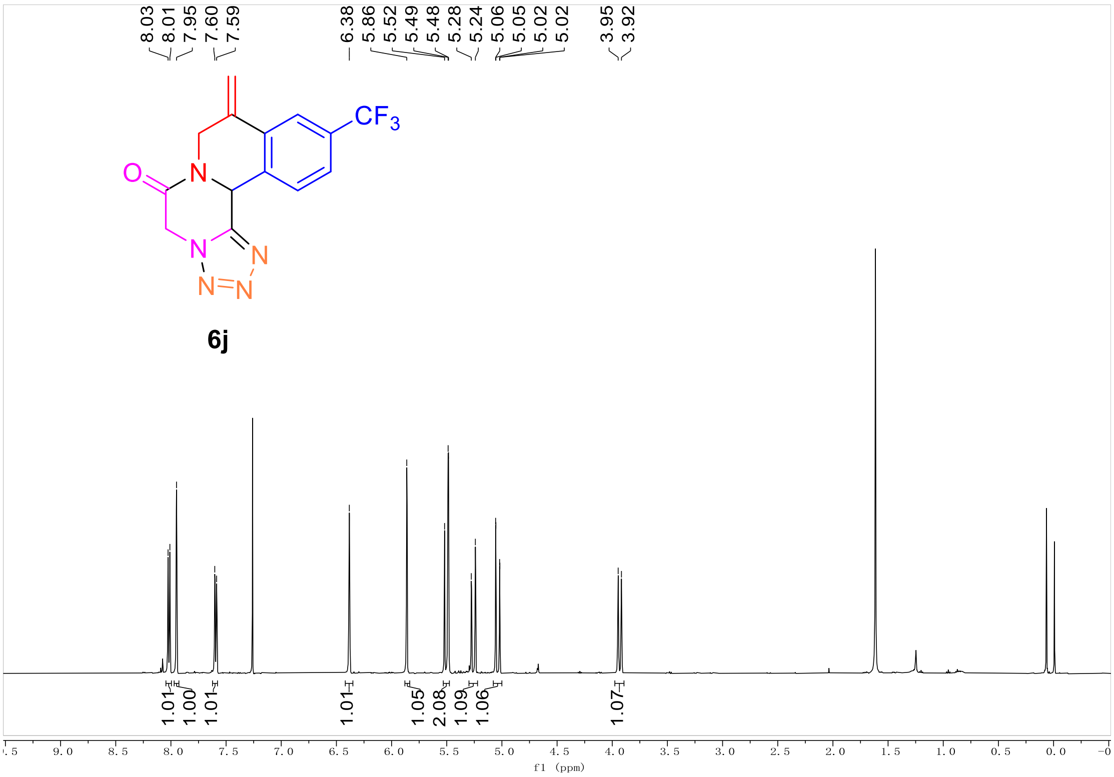
8.03 8.01 7.95 7.60 7.59 6.38 5.86 5.52 5.49 5.48 5.28 5.24 5.06 5.05 5.02 5.02 3.95 3.92
CF3
6j
1.01 1.00 1.01 1.01 1.05 2.08 1.09 1.06 1.07
f1 (ppm)

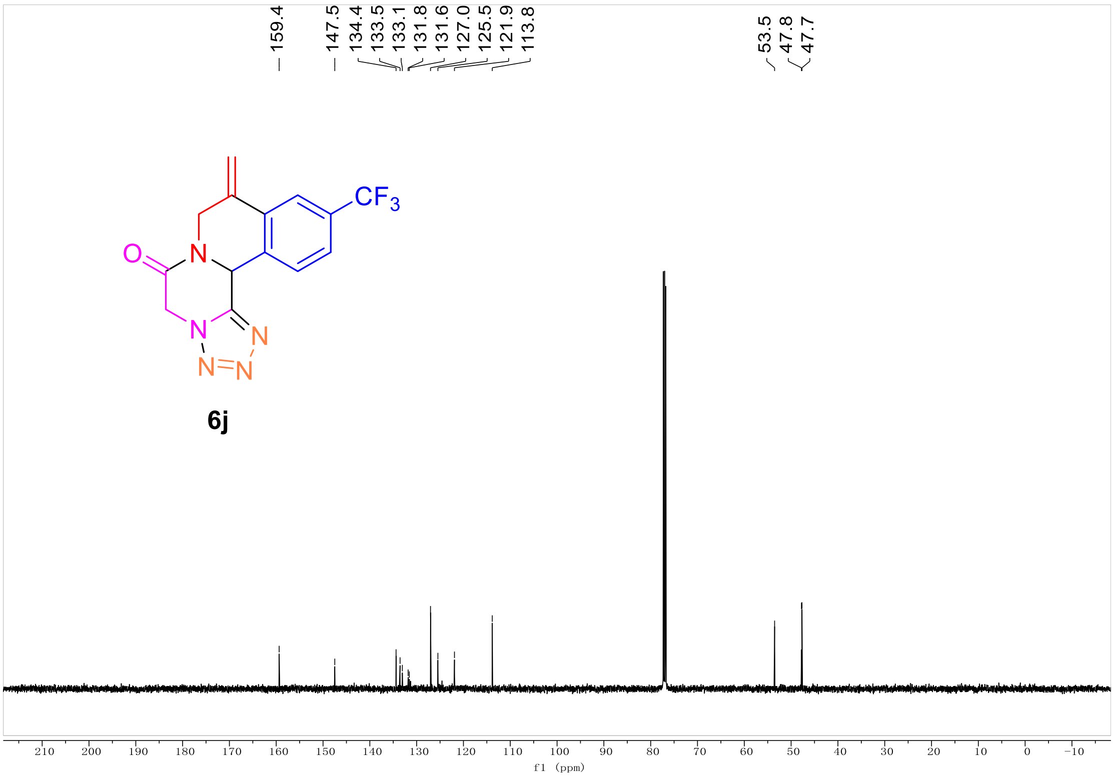
159.4 147.5 134.4 133.5 133.1 131.8 131.6 127.0 125.5 121.9 113.8 53.5 47.8 47.7
CF3
6j
f1 (ppm)

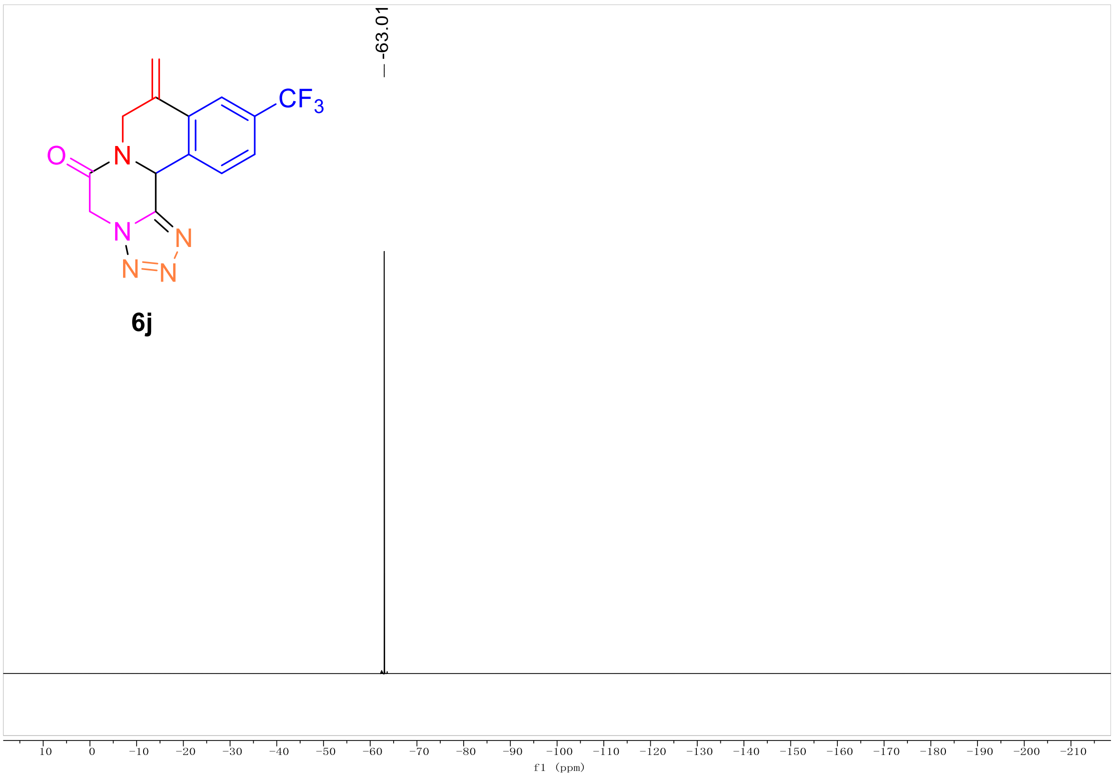
−63.01
CF3
O
N
N
N=N
6j
10 0 −10 −20 −30 −40 −50 −60 −70 −80 −90 −100 −110 −120 −130 −140 −150 −160 −170 −180 −190 −200 −210
f1 (ppm)

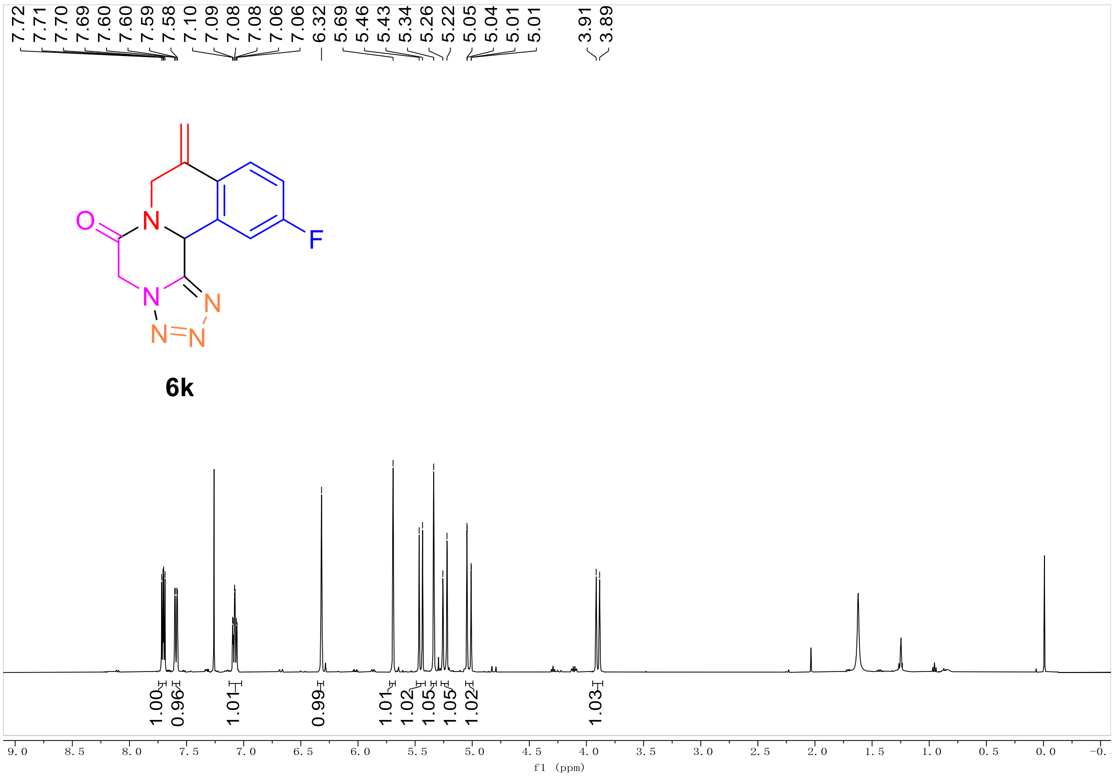
7.72 7.71 7.70 7.69 7.60 7.60 7.59 7.58 7.10 7.09 7.08 7.08 7.06 7.06 6.32 5.69 5.46 5.43 5.34 5.26 5.22 5.05 5.04 5.01 5.01 3.91 3.89
O
N
F
N
N=N
6k
1.00 0.96 1.01 0.99 1.01 1.02 1.05 1.05 1.02 1.03
9.0 8.5 8.0 7.5 7.0 6.5 6.0 5.5 5.0 4.5 4.0 3.5 3.0 2.5 2.0 1.5 1.0 0.5 0.0
f1 (ppm)

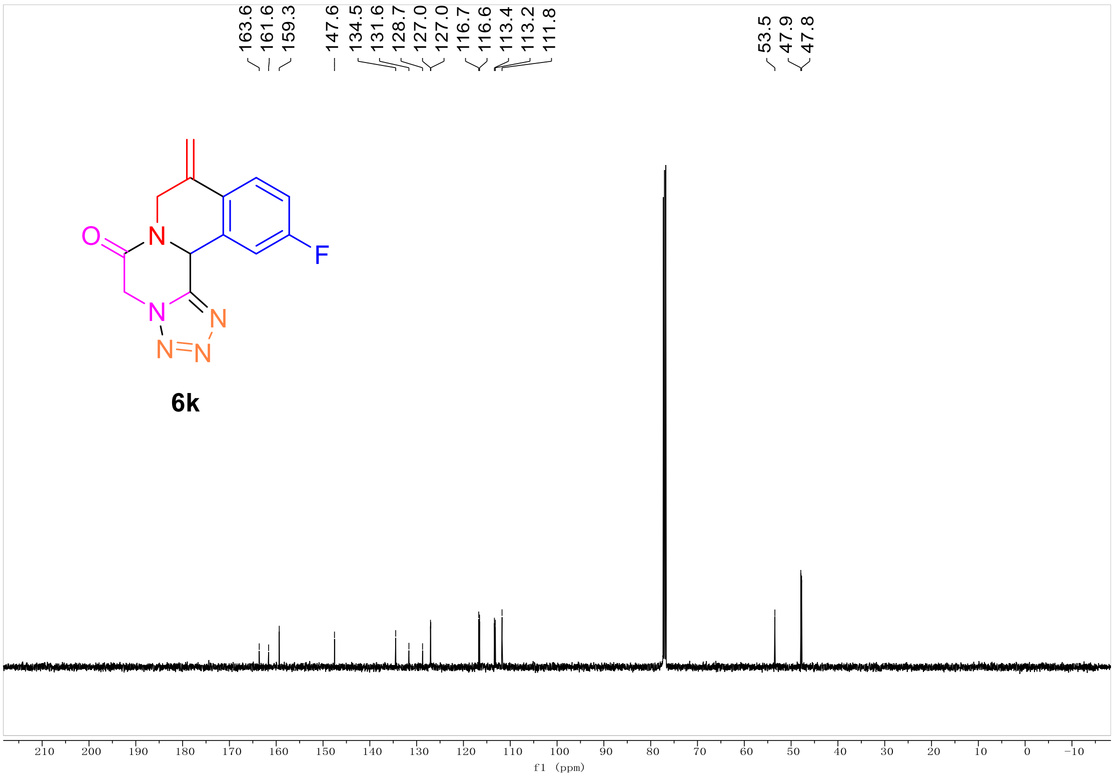
163.6
161.6
159.3
147.6
134.5
131.6
128.7
127.0
127.0
116.7
116.6
113.4
113.2
111.8
53.5
47.9
47.8
O
N
F
N
N
N
N
6k
f1 (ppm)

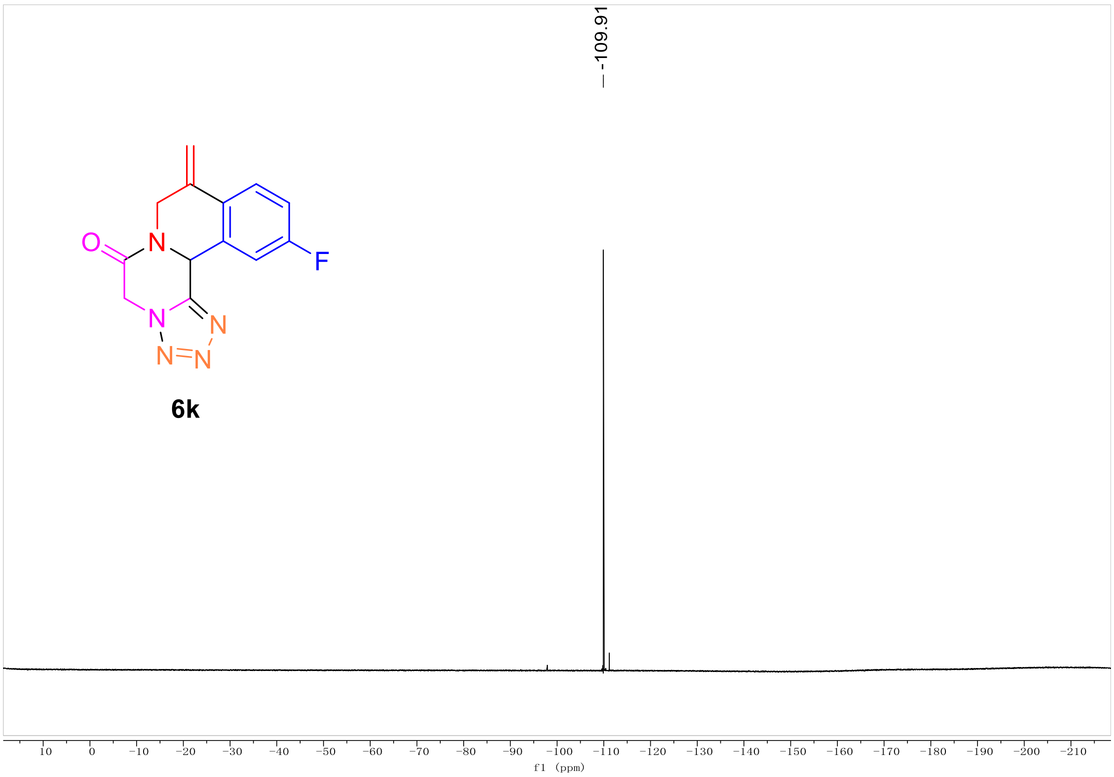
-109.91
O
N
F
N
N
N
N
6k
f1 (ppm)

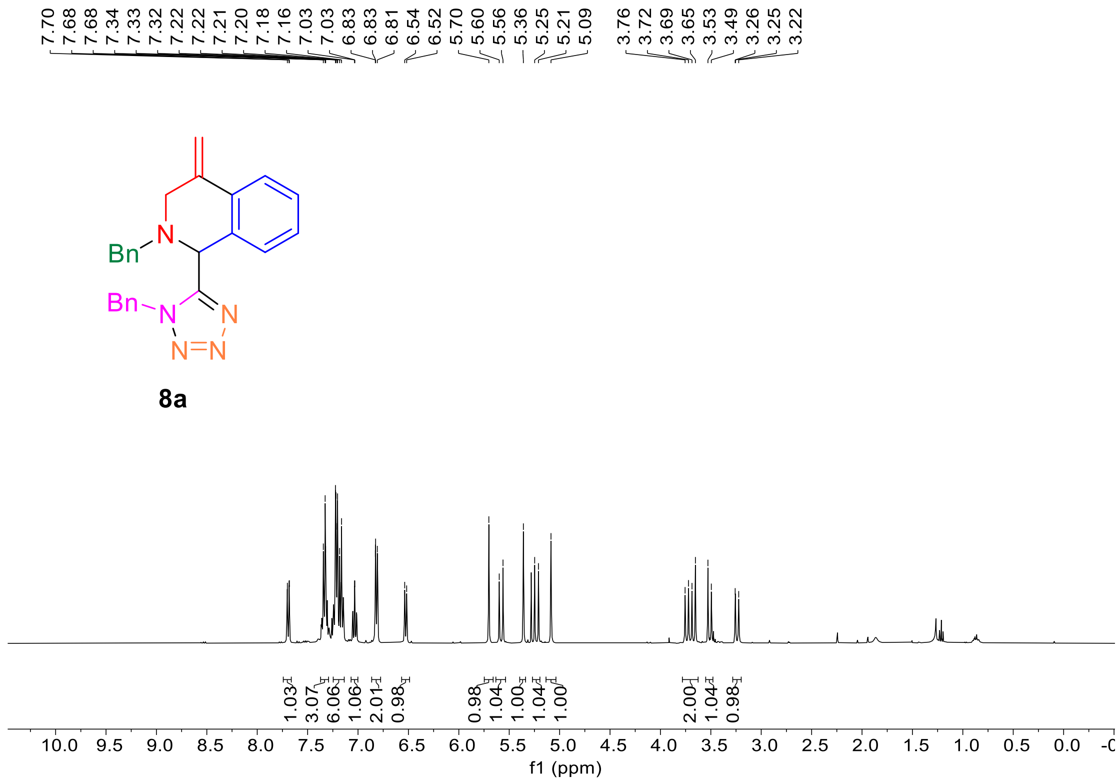
7.70
7.68
7.68
7.34
7.33
7.32
7.22
7.22
7.21
7.20
7.18
7.16
7.03
7.03
6.83
6.81
6.54
6.52
5.70
5.60
5.56
5.36
5.25
5.21
5.09
3.76
3.72
3.69
3.65
3.53
3.49
3.26
3.25
3.22
Bn
N
Bn
N
N
N=N
8a
1.03
3.07
6.06
1.06
2.01
0.98
0.98
1.04
1.00
1.04
1.00
2.00
1.04
0.98
f1 (ppm)

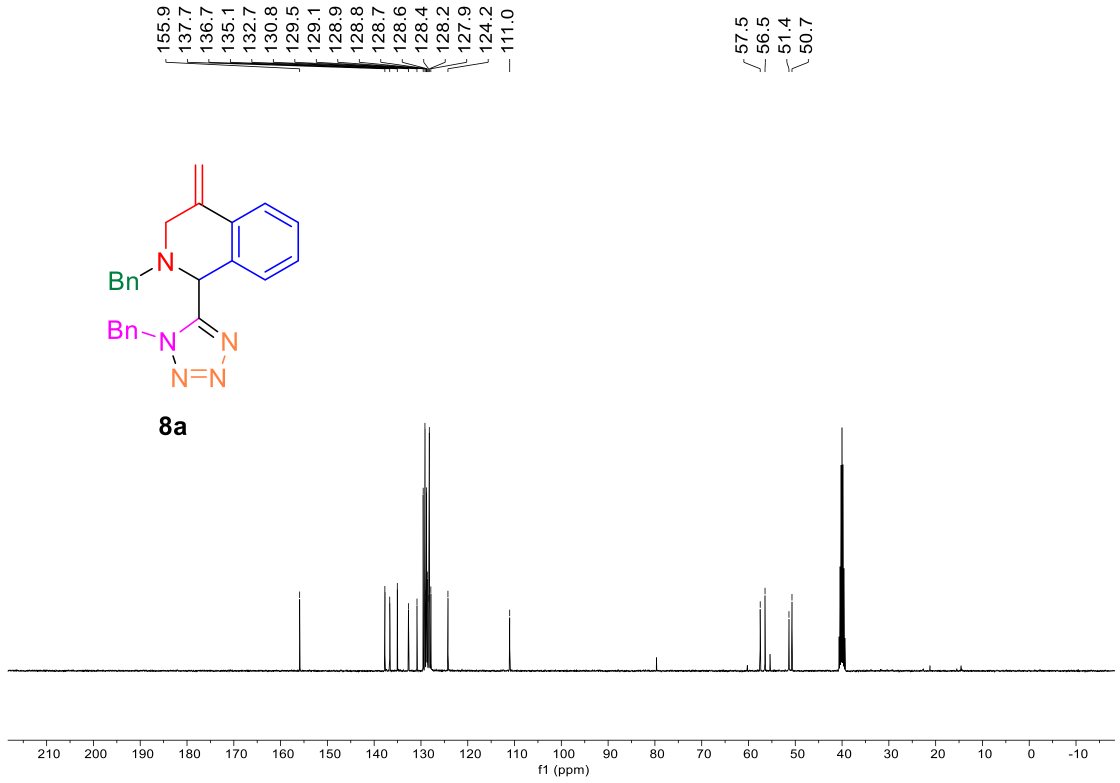
155.9
137.7
136.7
135.1
132.7
130.8
129.5
129.1
128.9
128.8
128.7
128.6
128.4
128.2
127.9
124.2
111.0
57.5
56.5
51.4
50.7
Bn
N
Bn
N
N
N=N
8a
f1 (ppm)

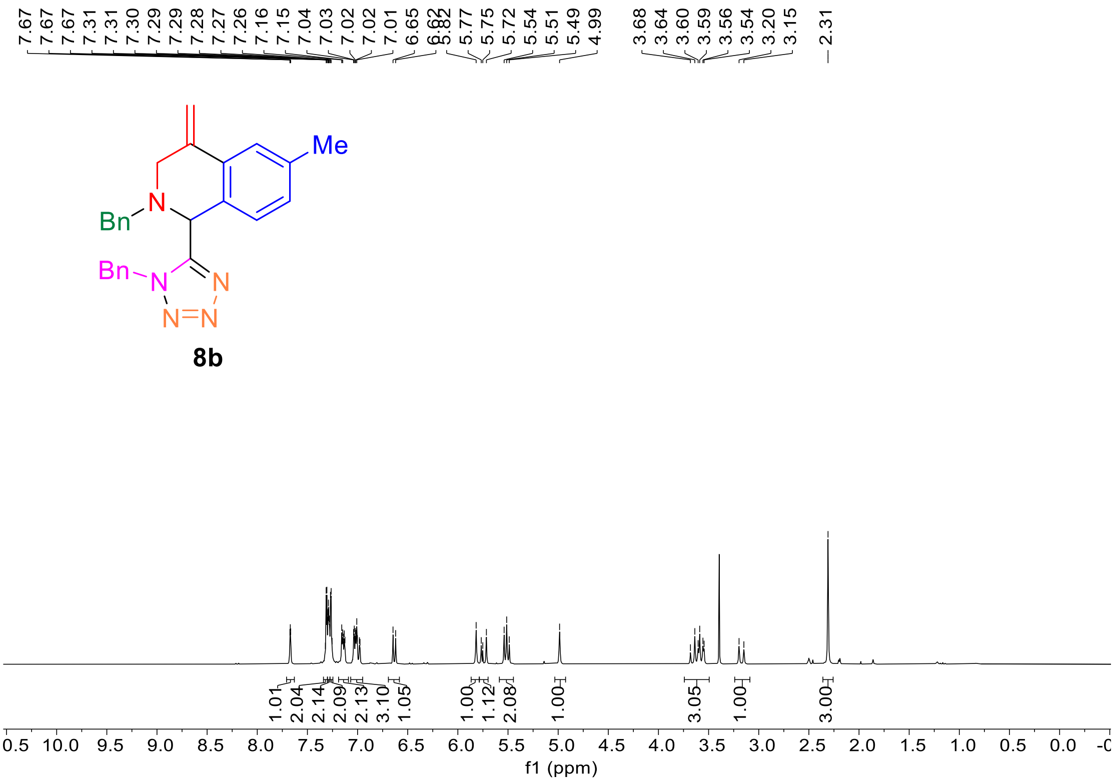
7.67 7.67 7.67 7.31 7.31 7.30 7.29 7.29 7.28 7.27 7.26 7.16 7.15 7.04 7.03 7.02 7.02 7.01 6.65 6.62 5.82 5.77 5.75 5.72 5.54 5.51 5.49 4.99 3.68 3.64 3.60 3.59 3.56 3.54 3.20 3.15 2.31
Me
Bn
N
Bn
N
N
N=N
8b
1.01 2.04 2.14 2.09 2.13 3.10 1.05 1.00 1.12 2.08 1.00 3.05 1.00 3.00
f1 (ppm)

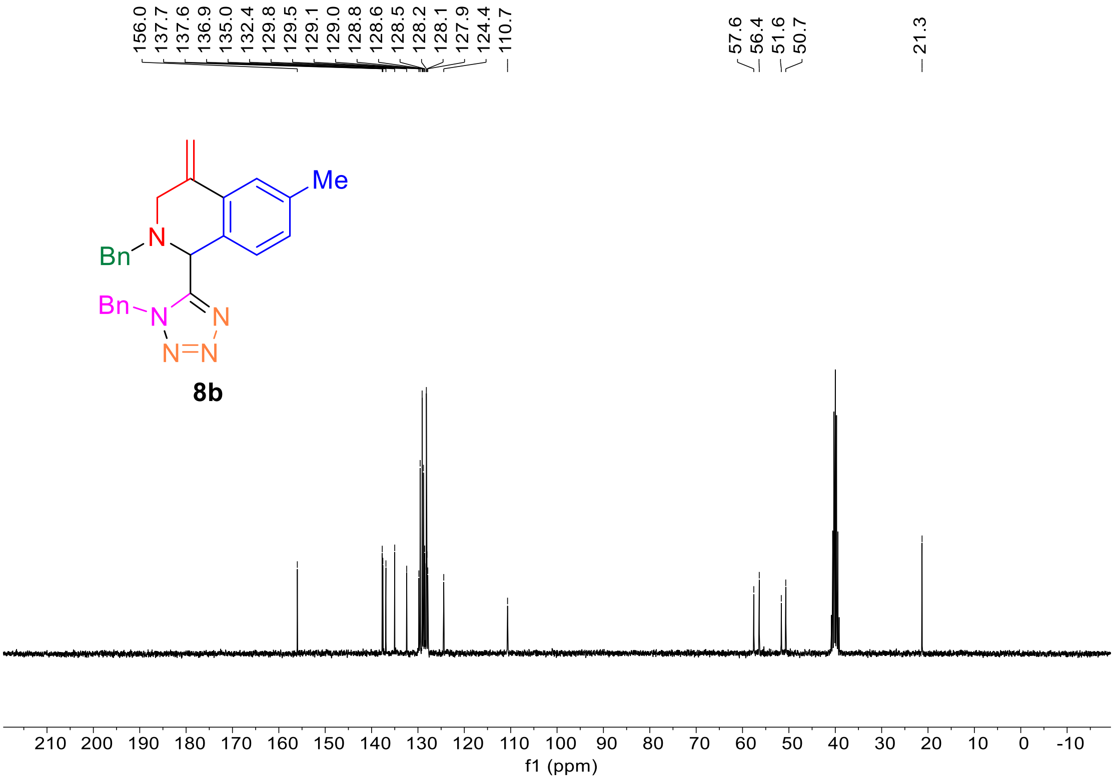
156.0 137.7 137.6 136.9 135.0 132.4 129.8 129.5 129.1 129.0 128.8 128.6 128.5 128.2 128.1 127.9 124.4 110.7 57.6 56.4 51.6 50.7 21.3
Me
Bn
N
Bn
N
N
N=N
8b
f1 (ppm)

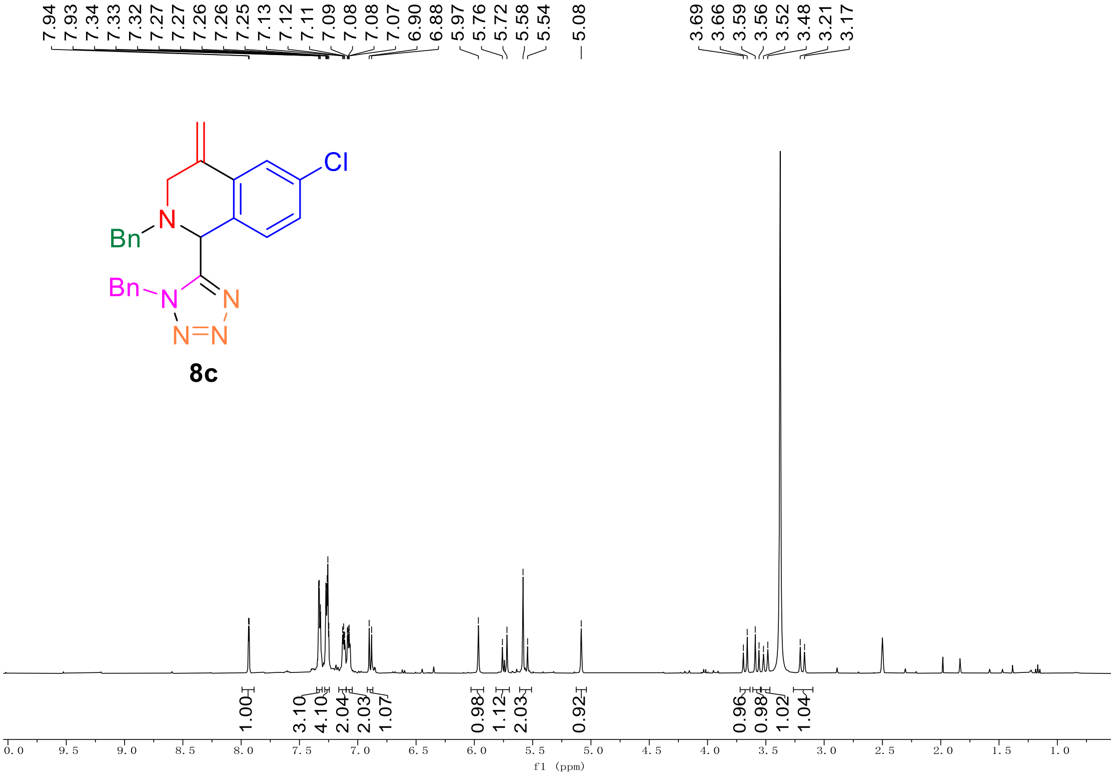
7.94 7.93 7.34 7.33 7.32 7.27 7.27 7.26 7.26 7.25 7.13 7.12 7.11 7.09 7.08 7.08 7.07 6.90 6.88 5.97 5.76 5.72 5.58 5.54 5.08
3.69 3.66 3.59 3.56 3.52 3.48 3.21 3.17
Cl
Bn
N
Bn
N
N
N=N
8c
1.00 3.10 4.10 2.04 2.03 1.07 0.98 1.12 2.03 0.92 0.96 0.98 0.98 1.02 1.04
f1 (ppm)

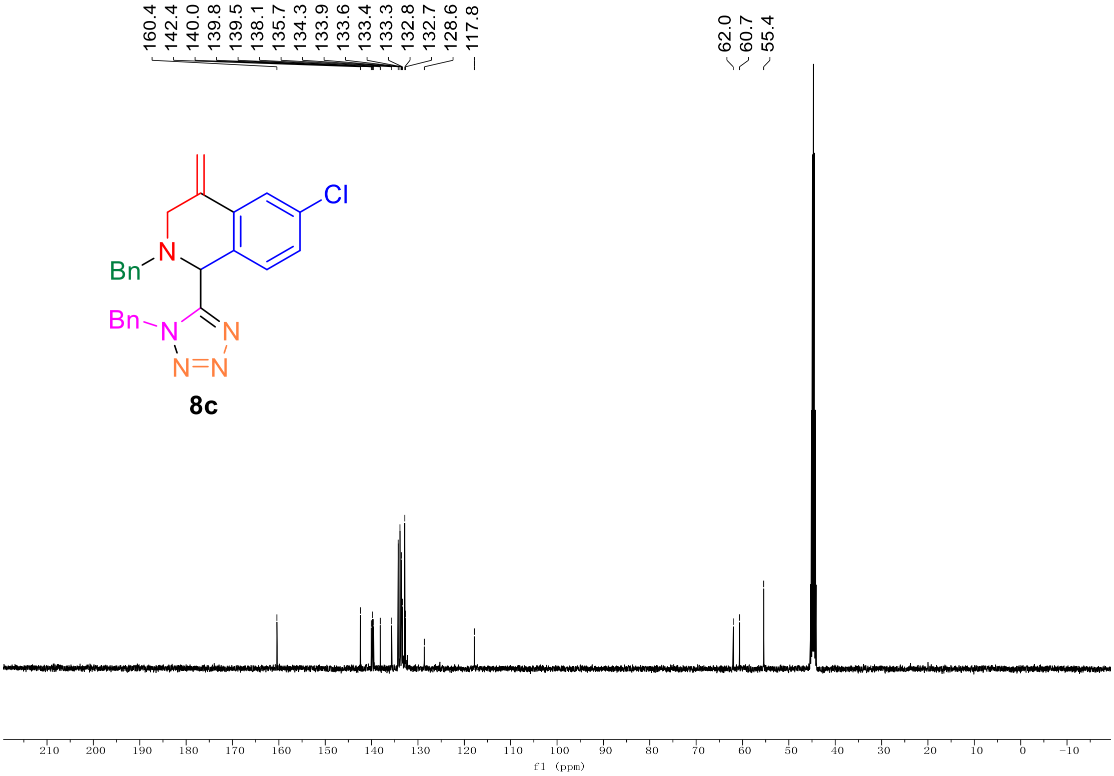
160.4 142.4 140.0 139.8 139.5 138.1 135.7 134.3 133.9 133.6 133.4 133.3 132.8 132.7 128.6 117.8
62.0 60.7 55.4
Cl
Bn
N
Bn
N
N
N=N
8c
f1 (ppm)

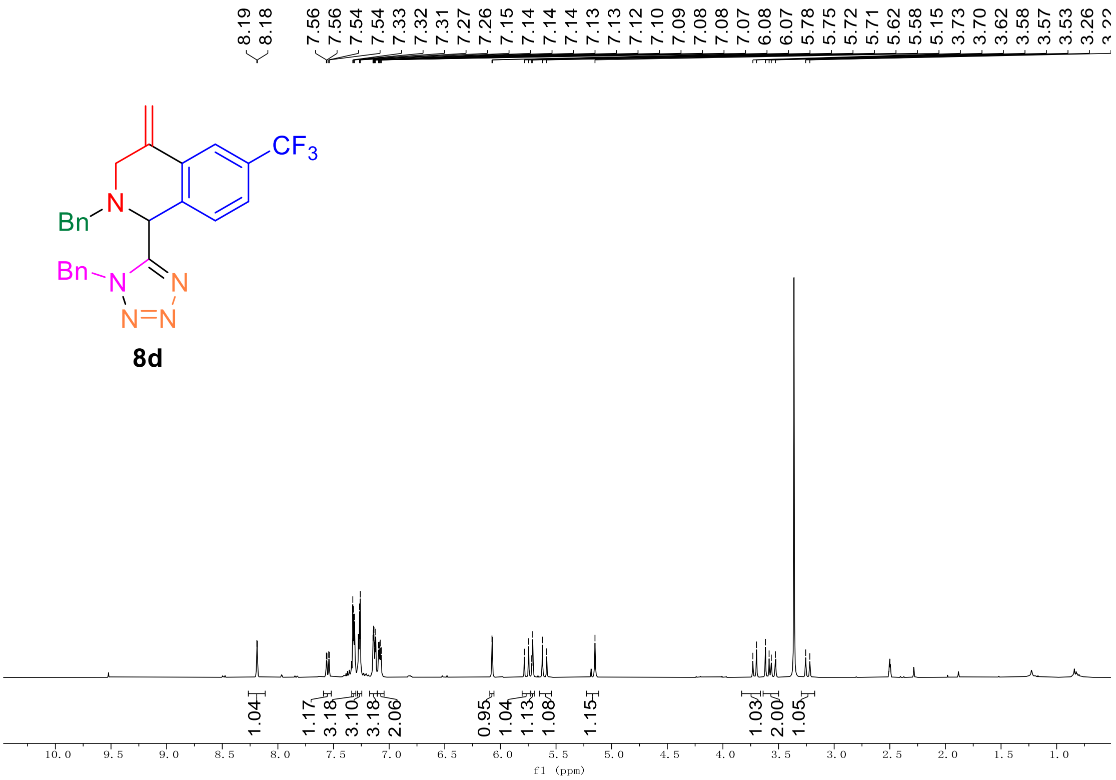
8.19
8.18
7.56
7.56
7.54
7.54
7.33
7.32
7.31
7.27
7.26
7.15
7.14
7.14
7.14
7.13
7.13
7.12
7.10
7.09
7.08
7.08
7.07
6.08
6.07
5.78
5.75
5.72
5.71
5.62
5.58
5.15
3.73
3.70
3.62
3.58
3.57
3.53
3.26
CF3
N
Bn
Bn
N
N
N=N
8d
1.04
1.17
3.18
3.10
3.18
2.06
0.95
1.04
1.13
1.08
1.15
1.03
2.00
1.05
f1 (ppm)

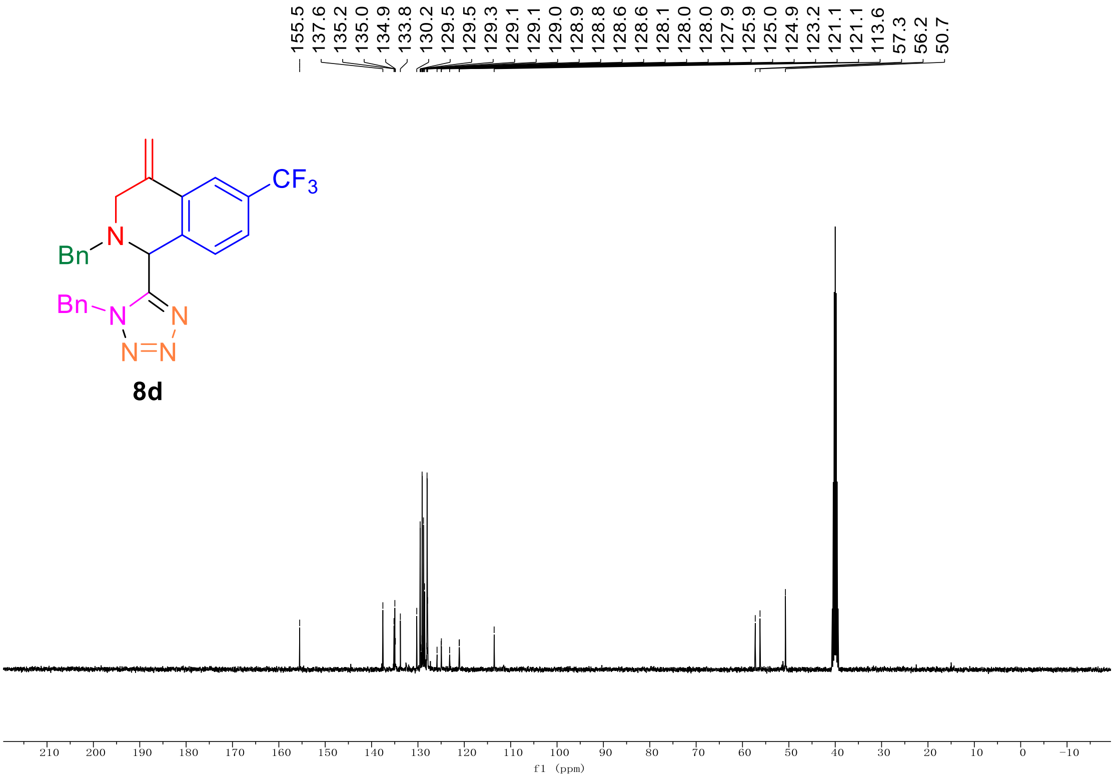
155.5
137.6
135.2
135.0
134.9
133.8
130.2
129.5
129.5
129.3
129.1
129.1
129.0
128.9
128.8
128.6
128.6
128.1
128.0
128.0
127.9
125.9
125.0
124.9
123.2
121.1
121.1
113.6
57.3
56.2
50.7
CF3
N
Bn
Bn
N
N
N=N
8d
f1 (ppm)

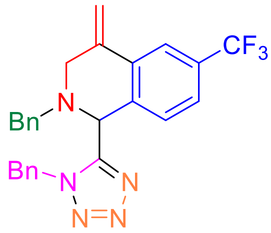
CF3
Bn
N
Bn
N
N=N
N

**8d**

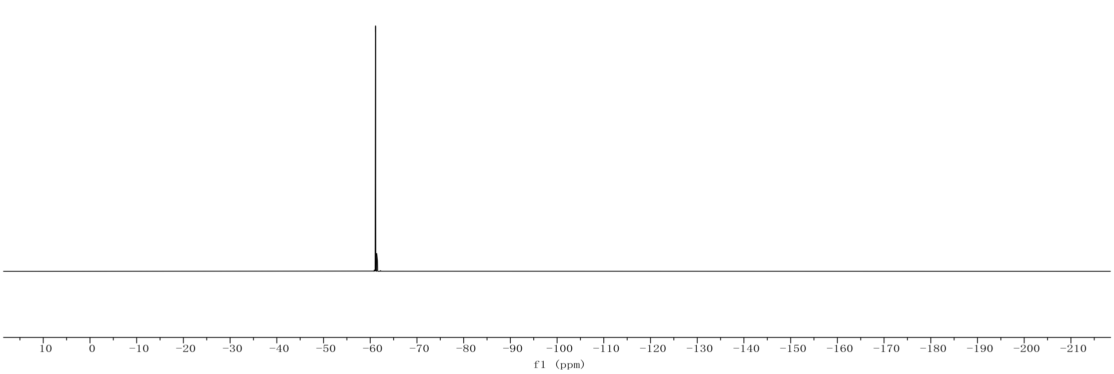
-61.13
10 0 -10 -20 -30 -40 -50 -60 -70 -80 -90 -100 -110 -120 -130 -140 -150 -160 -170 -180 -190 -200 -210
f1 (ppm)

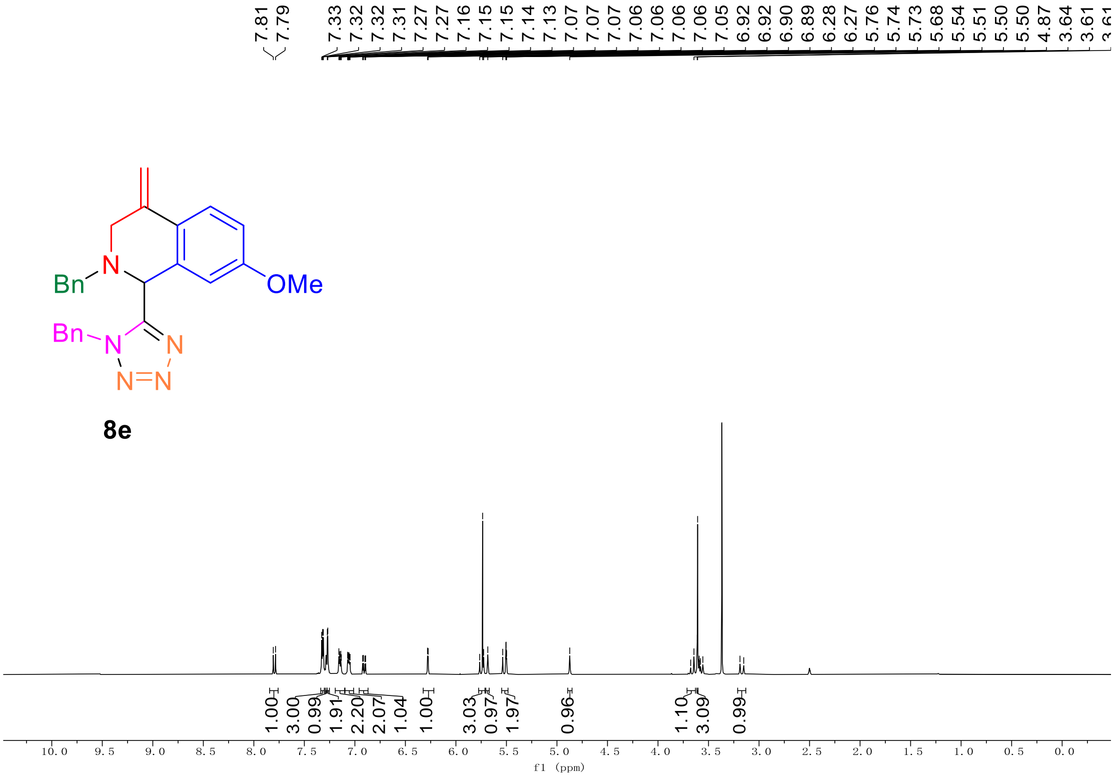
7.81
7.79
7.33
7.32
7.32
7.31
7.27
7.27
7.16
7.15
7.15
7.14
7.13
7.07
7.07
7.06
7.06
7.06
7.06
7.05
6.92
6.92
6.90
6.89
6.28
6.27
5.76
5.74
5.73
5.68
5.54
5.51
5.50
5.50
4.87
3.64
3.61
Bn
N
OMe
Bn
N
N
N=N
8e
1.00
3.00
0.99
1.91
2.20
2.07
1.04
1.00
3.03
0.97
1.97
0.96
1.10
3.09
0.99
f1 (ppm)

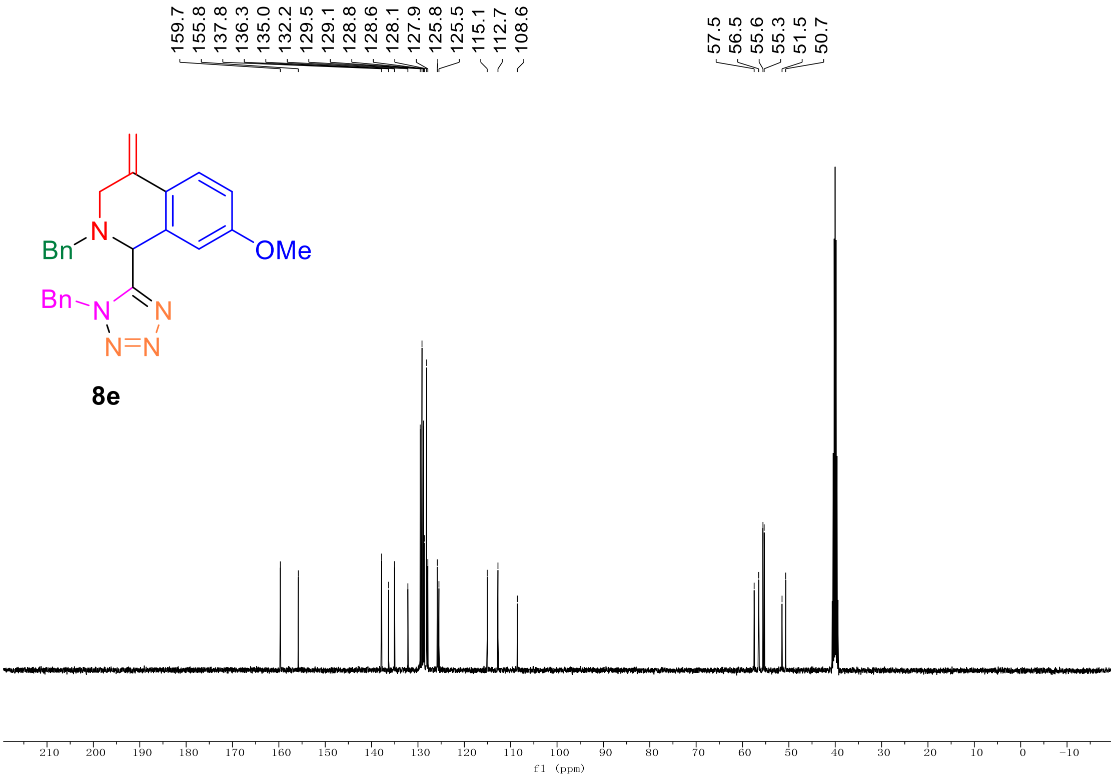
159.7
155.8
137.8
136.3
135.0
132.2
129.5
129.1
128.8
128.6
128.1
127.9
125.8
125.5
115.1
112.7
108.6
57.5
56.5
55.6
55.3
51.5
50.7
Bn
N
OMe
Bn
N
N
N=N
8e
f1 (ppm)

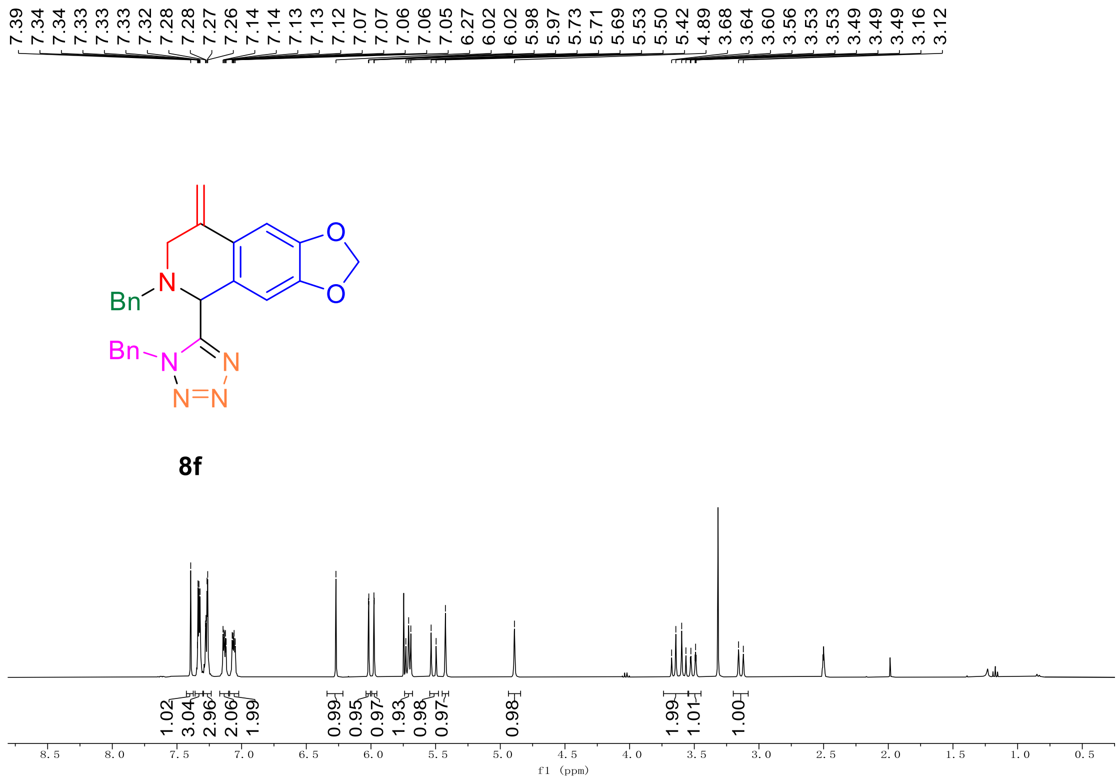
7.39 7.34 7.34 7.33 7.33 7.32 7.28 7.28 7.27 7.26 7.14 7.14 7.13 7.13 7.12 7.07 7.07 7.06 7.06 7.05 6.27 6.02 6.02 5.98 5.97 5.73 5.71 5.69 5.53 5.50 5.42 4.89 3.68 3.64 3.60 3.56 3.53 3.53 3.49 3.49 3.49 3.16 3.12
Bn
N
O
O
Bn
N
N
N=N
8f
1.02 3.04 2.96 2.06 1.99 0.99 0.95 0.97 1.93 0.98 0.97 0.98 1.99 1.01 1.00
f1 (ppm)

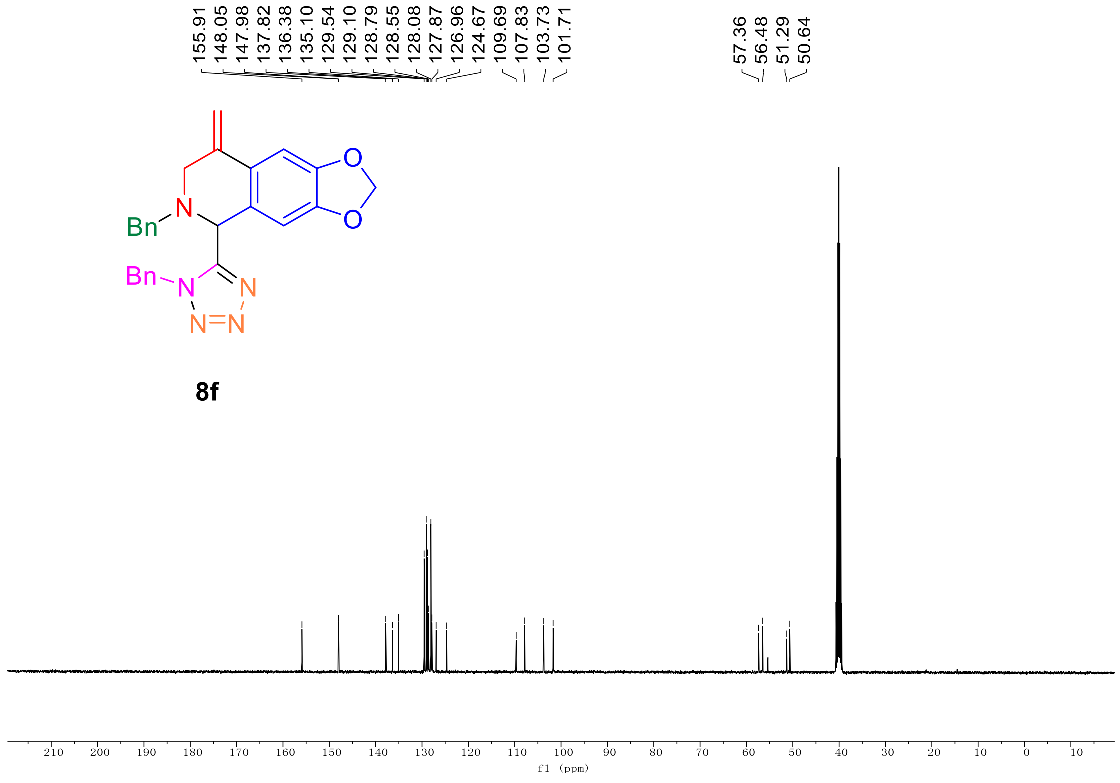
155.91 148.05 147.98 137.82 136.38 135.10 129.54 129.10 128.79 128.55 128.08 127.87 126.96 124.67 109.69 107.83 103.73 101.71
57.36 56.48 51.29 50.64
Bn
N
O
O
Bn
N
N
N=N
8f
f1 (ppm)

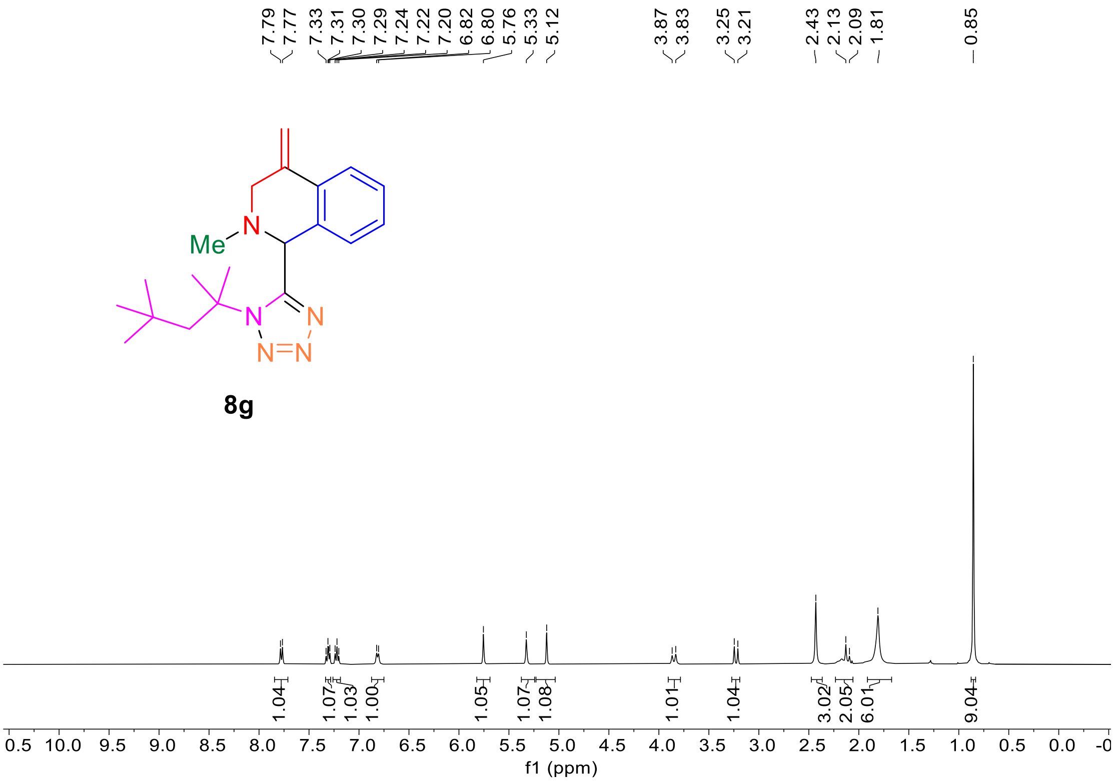
7.79 7.77 7.33 7.31 7.30 7.29 7.24 7.22 7.20 6.82 6.80 5.76 5.33 5.12 3.87 3.83 3.25 3.21 2.43 2.13 2.09 1.81 0.85
Me
N
8g
1.04 1.07 1.03 1.00 1.05 1.07 1.08 1.01 1.04 3.02 2.05 6.01 9.04
f1 (ppm)

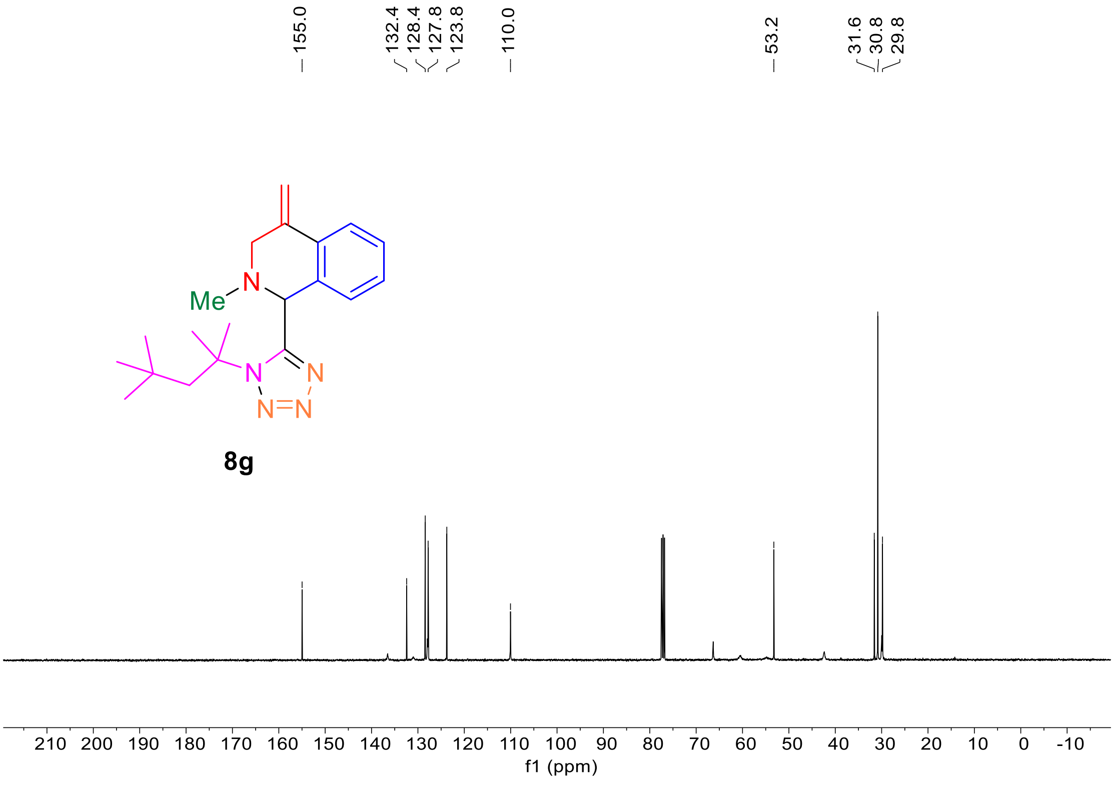
155.0 132.4 128.4 127.8 123.8 110.0 53.2 31.6 30.8 29.8
Me
N
8g
f1 (ppm)

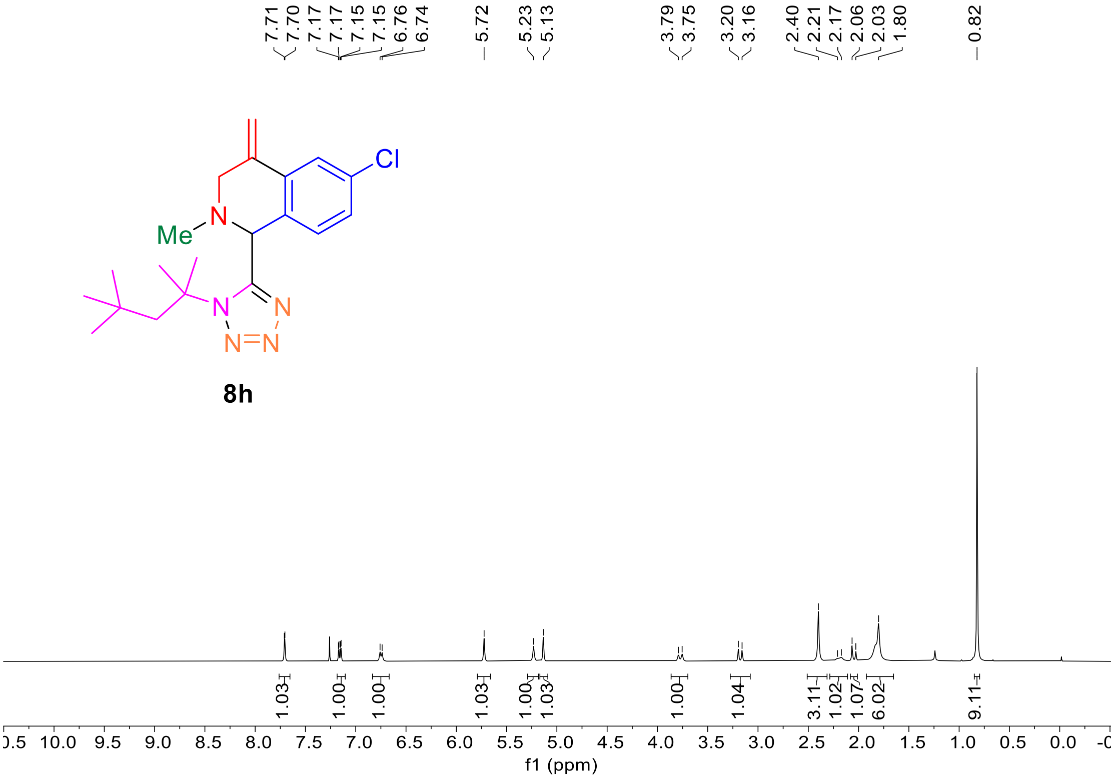
7.71 7.70 7.17 7.17 7.15 7.15 6.76 6.74
5.72
5.23 5.13
3.79 3.75
3.20 3.16
2.40 2.21 2.17 2.06 2.03 1.80
0.82
Cl
Me
N
8h
1.03 1.00 1.00 1.03 1.00 1.03 1.00 1.04 3.11 1.02 1.07 6.02 9.11
f1 (ppm)

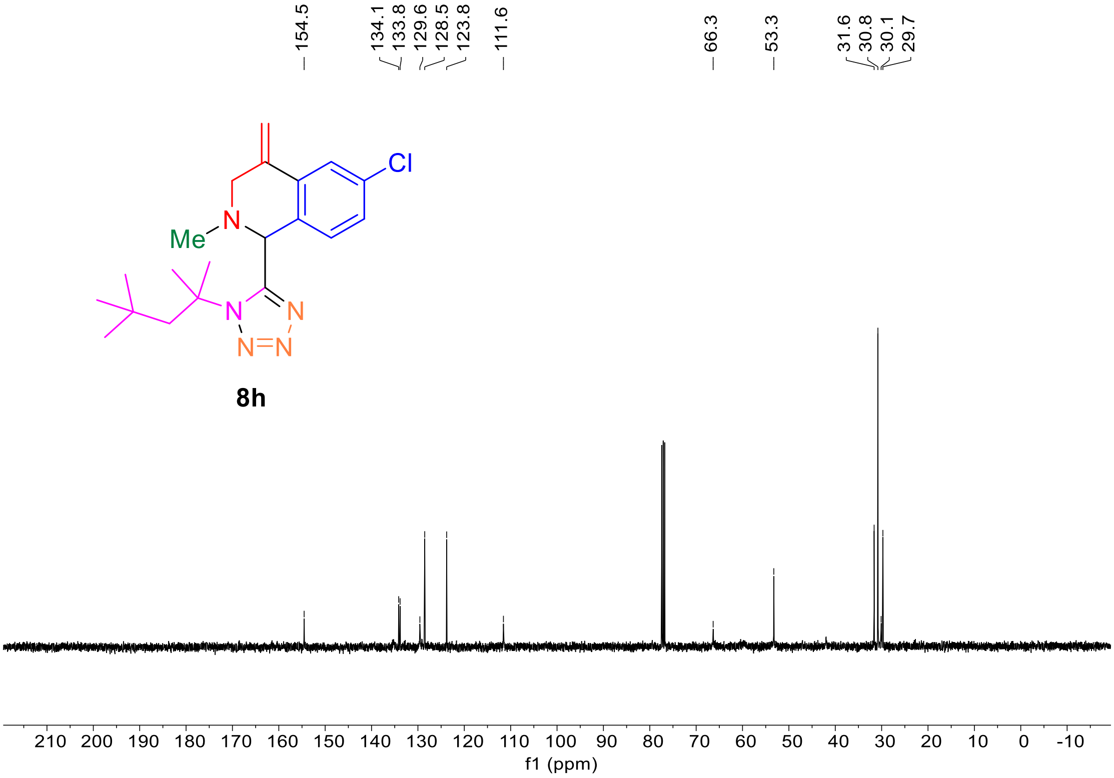
154.5
134.1 133.8 129.6 128.5 123.8
111.6
66.3
53.3
31.6 30.8 30.1 29.7
Cl
Me
N
8h
f1 (ppm)

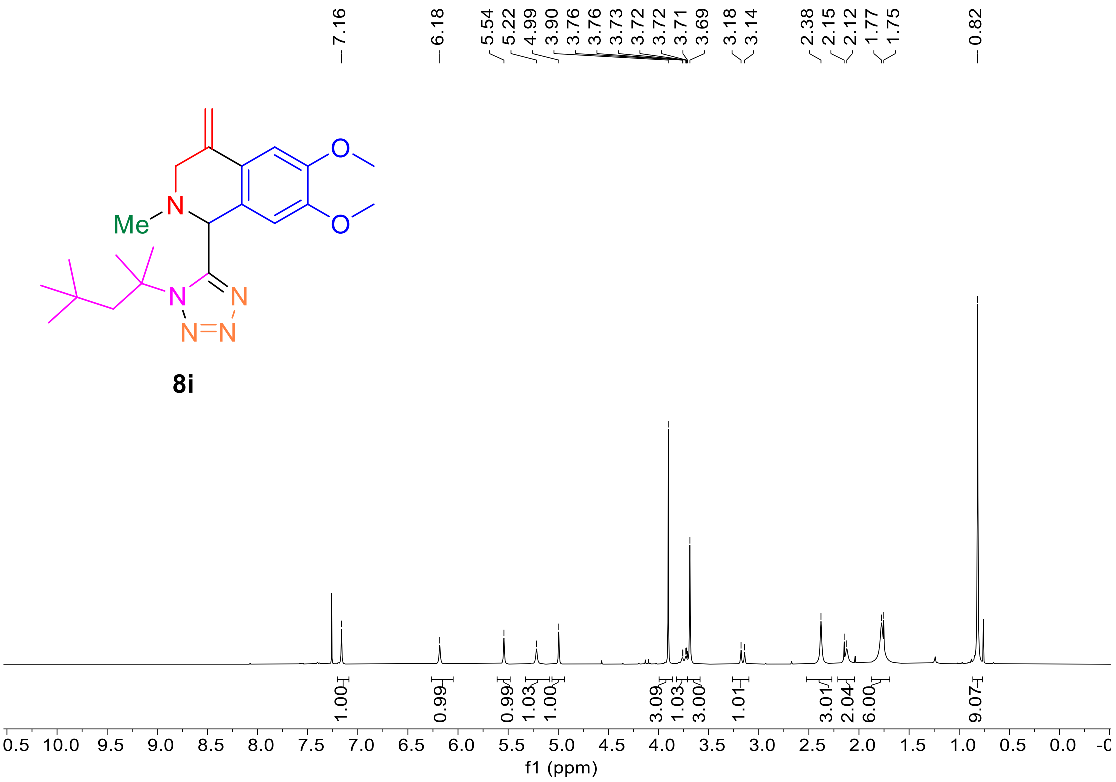
7.16
6.18
5.54
5.22
4.99
3.90
3.76
3.76
3.73
3.72
3.72
3.71
3.69
3.18
3.14
2.38
2.15
2.12
1.77
1.75
0.82
Me
N
O
O
N
N=N
N
8i
1.00
0.99
0.99
1.03
1.00
3.09
1.03
3.00
1.01
3.01
2.04
6.00
9.07
f1 (ppm)

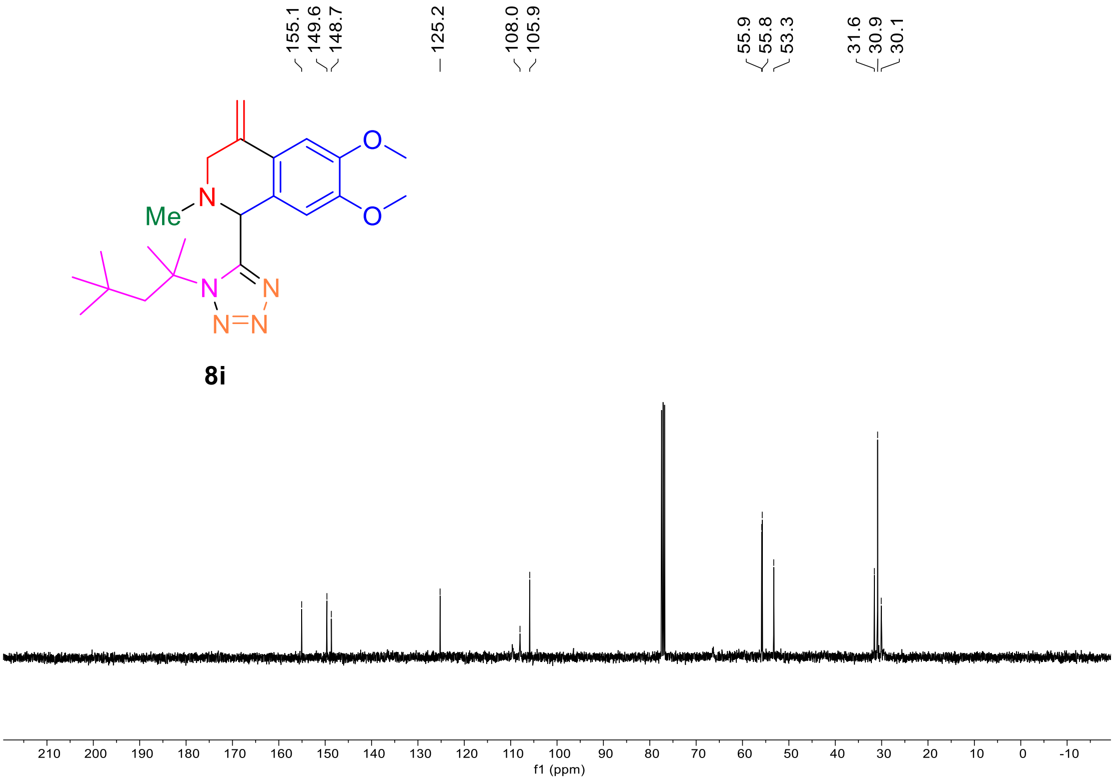
155.1
149.6
148.7
125.2
108.0
105.9
55.9
55.8
53.3
31.6
30.9
30.1
Me
N
O
O
N
N=N
N
8i
f1 (ppm)

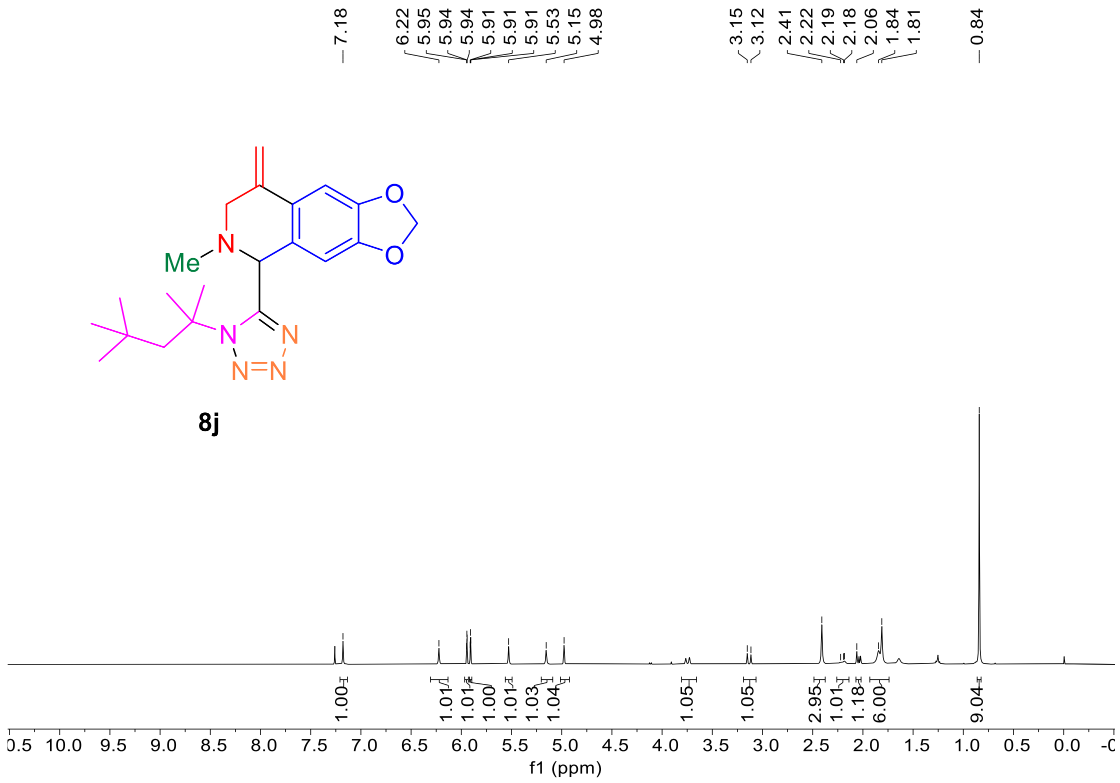
7.18
6.22 5.95 5.94 5.94 5.91 5.91 5.91 5.53 5.15 4.98
3.15 3.12
2.41 2.22 2.19 2.18 2.06 1.84 1.81
0.84
Me
N
O
N=N
8j
1.00 1.01 1.01 1.00 1.01 1.03 1.04 1.05 1.05 2.95 1.01 1.18 6.00 9.04
f1 (ppm)

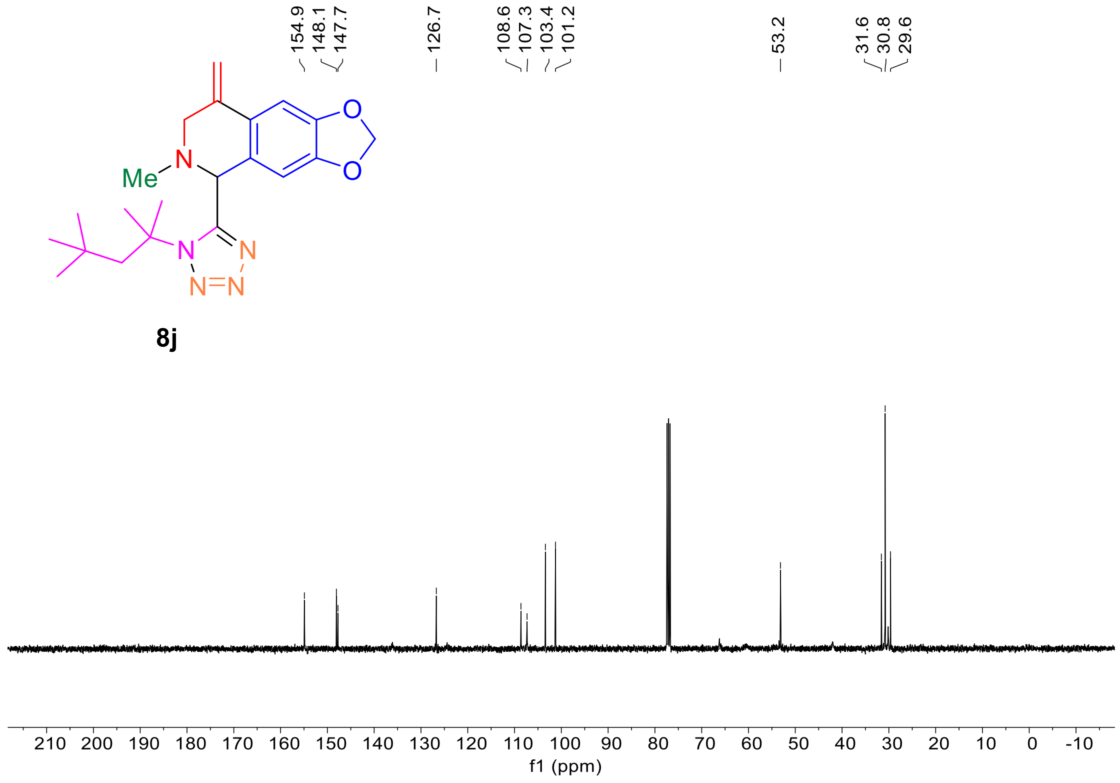
154.9 148.1 147.7
126.7
108.6 107.3 103.4 101.2
53.2
31.6 30.8 29.6
Me
N
O
N=N
8j
f1 (ppm)
